# Photoelectrochemical glycerol oxidation on Mo-BiVO_4_ photoanodes shows high photocharging current density and enhanced H_2_ evolution†

**DOI:** 10.1039/d2ya00077f

**Published:** 2022-08-19

**Authors:** Debajeet K. Bora, Manouchehr Nadjafi, Andac Armutlulu, Davood Hosseini, Pedro Castro-Fernández, Rita Toth

**Affiliations:** Laboratory of Energy Science and Engineering, Institute for Energy Technology, ETH Zurich CH-8092 Zurich Switzerland debajeet.bora@um6p.ma; Laboratory for High-Performance Ceramics, Empa. Swiss Federal Laboratories for Materials Science and Technology Überlandstrasse 129 CH-8600 Dübendorf Switzerland

## Abstract

Mo-doped BiVO_4_'s lower efficiency can be attributed in part to exciton recombination losses. Recombination losses during photoelectrochemical water oxidation can be eliminated by using glycerol as a hole acceptor. This results in an enhanced photocurrent density. In this research, we present the synthesis of a Mo-doped BiVO_4_ photoelectrode with a greater photocurrent density than a traditional pristine photoanode system. Increased photon exposure duration in the presence of glycerol leads to 8 mA cm^−2^ increase in photocurrent density due to the creation of a capacitance layer and a decrease in charge transfer resistance on the photoelectrode in a neutral-phosphate buffer solution thus confirming the photo charging effect. Glycerol photooxidation improves the photoelectrode's rate of hydrogen evolution. Research into the effects of electrolyte and electrode potential on photoelectrodes has revealed that when the applied potential increases, the light absorbance behaviour changes following its absorption distribution over the applied potential. Under a transmission electron microscope (TEM), a unique dynamical crystal fringe pattern is found in the nanoparticles scratched from the photoelectrode.

## Abstract 2

For enhancing hydrogen evolution, solution-processed Mo-BiVO_4_ exhibits a distinct atomic phenomenon with a high photocharging current density.

## Graphical abstract

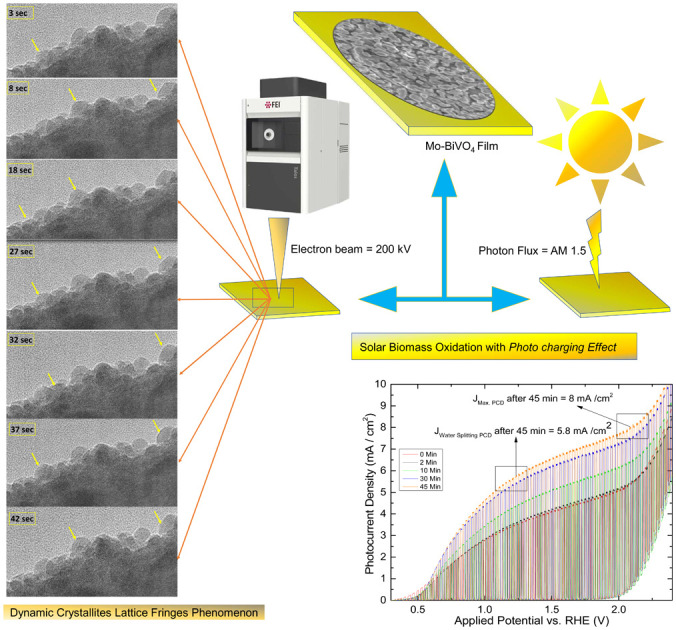

## Introduction

The application of n-type metal oxide photoelectrodes for photoelectrochemical solar water splitting to generate hydrogen has been a cornerstone of artificial photosynthesis research over the last decade.^1–3^ Numerous binary and ternary metal oxide photoelectrodes are being developed to meet the criteria for the most efficient materials in terms of solar to hydrogen conversion (STH) and incident photon to current conversion (IPCE) efficiencies.^4^ In this regard, the use of BiVO_4_ as a highly efficient photoanode material with a theoretical STH efficiency of 9.1% is an intriguing prospect.^5^ It is widely used in photocatalysis and photoelectrochemical water splitting for hydrogen production due to its low bandgap and visible light absorbing nature. BiVO_4_ was used in this case for photoelectrochemical water splitting in a variety of morphological forms, including nanocrystalline, nanowire, and nano porous.^6–12^ Due to one of its primary limitations in terms of charge transport *via* self-trapped charge carriers known as small polarons, higher valence metal ion doping is typically preferred.^13,14^ Tungsten (W), for example, enhances the performance of a Co-pi treated W-doped BiVO_4_ photoelectrode.^15^ Molybdenum (Mo) and W co-doping increases the photocurrent density tenfold when compared to undoped BiVO_4_. By altering the surface passivation trap layer and recombination centers at grain boundaries and interfaces, doping aids in the separation of electron-hole pairs^16^ as well as electron transport.^17^ Mo-doping also does not affect the electrode's bandgap or light-harvesting capability.^5^ According to DFT calculations, Mo atoms prefer to substitute for V atoms in the bulk phase, and charge carriers can be effectively accelerated as a result. Mo atoms replace Bi atoms on the surface, resulting in a surface oxygen quasi-vacancy. This aids in the exposure of Bi atoms to water molecules for adsorption.^18^ X-ray absorption spectroscopy reveals that W dopants occupy the V sites, resulting in a less distorted local structure at the Bi centre, while transient absorption spectroscopy reveals a significant alteration of hole traps, inhibiting carrier recombination and increasing electron lifetime.^19^ Apart from the doping strategy, it was found that the heterojunction type of BiVO_4_ devices increased the overall efficiency. In this context, the heterojunction refers to the interaction of two metal oxide layers. For instance, a doping study of Cr^6+^, W^6+^, and Mo^6+^ found that Mo^6+^ is critical for increasing the charge transfer efficiency. To supplement this, an additional WO_3_ layer beneath the Mo-BiVO_4_ layer improves the charge separation efficiency by up to 50%.^20^ Gradient-based tungsten (W) doping has been used similarly to create homojunction devices in tungsten doped BiVO_4_. This results in an 80% carrier separation efficiency and a 4.9% STH conversion efficiency in a silicon solar cell tandem configuration.^21^ Additionally, a hematite-based hetero dual-type photoanode is being developed to increase the STH efficiency by up to 7% by coating it with a BiVO_4_ layer.^22^ Apart from charge transport, the light absorption properties of these materials are considered when heterojunction devices with nanoengineered structures are constructed. In this case, a cone-shaped electrode was developed to overcome the constraint imposed by the short carrier diffusion length on the thickness of Mo doped BiVO_4_ layers. This electrode converts STH at a rate of 6.2 per cent when used in conjunction with a perovskite solar cell.^23^ Another intriguing way to boost BiVO_4_'s efficacy is through its photo charging effect. It is achieved by repeatedly exposing AM1.5G to an open circuit. It increases the photocurrent's cathodic shift onset strength. Typically, the photo charging is carried out in a basic electrolyte; acidic media have no effect. Phosphate buffers exhibit issues with etching and stability. Prolonged exposure to BiVO_4_ in aqueous solution under open-circuit conditions improves the efficiency of solar water oxidation by lowering the onset potential and increasing the maximum photocurrent density.^24,25^

Recently, BiVO_4_ has been used to convert biomass into value-added organics. Due to its well-matched valence band edge (VB) position with the electrochemical oxidation potential of 5-hydroxymethyl furfuryl, it is used in the conversion to 2,5-furan dicarboxylic acid using TEMPO (2,2,6,6-teteramethylpyridine-1-oxyl) as an ion scavenger.^26^ Additionally, biomass is a critical component of the global energy landscape and has a direct effect on the energy market. Solar biomass splitting is a low-cost and energy-intensive alternative to biomass energy conversion (high temperature). As a by-product of the biodiesel industry, the biomass waste ‘Glycerol’ is produced in the range of 800k tons per year. It has the chemical formula C_3_H_8_O_3_ and readily adsorbs semiconductors with defects. Additionally, PEC biomass oxidation is a safe process because it does not involve oxygen co-evolution and requires less overpotential (glucose oxidation, −0.01 V) than PEC oxidation (1.23 V). PEC biomass oxidation is already demonstrated on Ti-doped hematite semiconductors.^27–31^ Recently, it was reported that the photoelectrochemical conversion of glycerol to value-added organics using pure BiVO_4_ achieved an 80% conversion rate to formic acid and dihydroxyacetone.^32–35^

The purpose of this work is to increase the photocurrent density to meet the current state of the art, as well as the efficiency of solar to hydrogen conversion and the faradaic efficiency for hydrogen formation. As described in a recently published work, PEC based glycerol oxidation is primarily used to produce value-added organics, with an emphasis on controlling the activity-selectivity factors of the BiVO_4_ photoanode.^32–35^ Additionally, the same methodology for hydrogen production is available in the current scenario,^32^ as hydrogen is produced as a by-product of CO_2_ and formic acid/formaldehyde formation on the cathodic side. It is extremely useful for removing the explosive mixture of H_2_/O_2_ caused by any type of membrane leakage. In terms of kinetics, PEC biomass oxidation is more advantageous than water oxidation because it involves fewer reaction intermediate steps, which increases the associated potential. The most critical research gap here is the direct application of photoelectrochemical biomass oxidation for increasing the solar to hydrogen conversion efficiency (STH) of existing Mo-BiVO_4_ without resorting to expensive modification technologies such as heterojunction formation or the tandem approach. The following unresolved issue in this regard is an understanding of the semiconductor–organic interface. Following that, no report on the crystallographic changes in BiVO_4_ caused by high energy TEM beam exposure is available. As BiVO_4_ is notorious for its photodecomposition reaction, the effect of high energy electron particles on its stability will shed light.

To address these questions, the current work aims to prepare a Mo-doped BiVO_4_ using a novel synthetic route that results in a nanostructured material. Following that, the crystallographic understanding of crystal fringe evolution will be examined to “shed light on its stability”. To increase the STH efficiency, a detailed understanding of PEC in the presence of glycerol will be discussed. Following that, we will discuss the operando Spectro electrochemical understanding required to deduce the absorbance pattern's relationship to the applied potential. To understand further the effect of PEC glycerol oxidation on semiconductors, we will conduct an *ex situ* investigation of the photoelectrode in the electrolyte (phosphate buffer) and glycerol using a variety of auxiliary characterization techniques, with particular emphasis on the Bi/V ratio and their oxidation state variation.

## Results and discussion

### Synthesis mechanism and fundamental studies of the Mo-BiVO_4_ photoelectrode concerning optical, crystallographic, morphological and photoelectrochemical properties

**Scheme 1:**
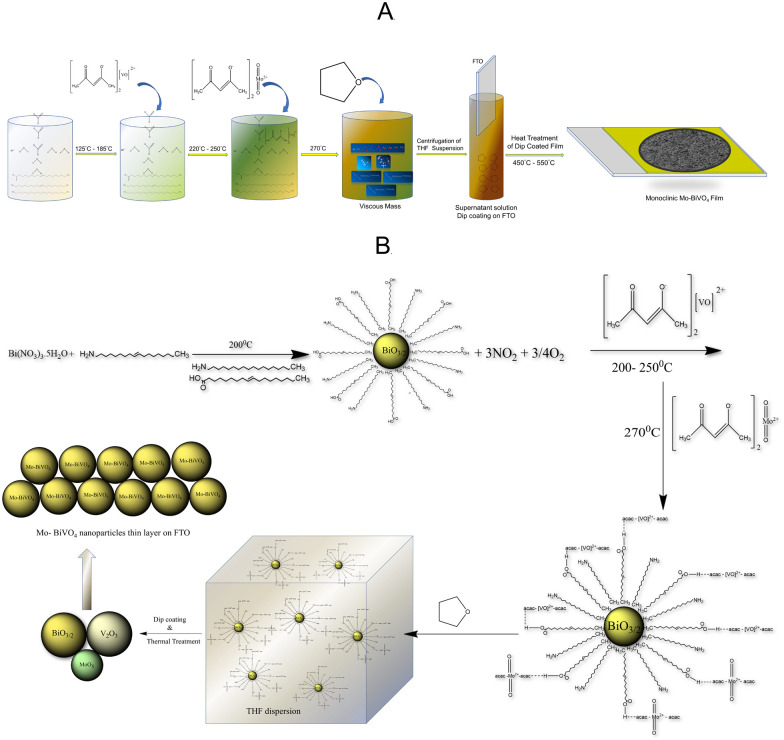
(A) Non-aqueous thermal decomposition of Mo-BiVO_4_ to produce a monoclinic photoelectrode. (B) The mechanism by which Mo-BiVO_4_ is formed from stoichiometric BiO_3/2_ particles capped with metal acetylacetonate derivatives of oleic acid *via* weak hydrogen bonding.

The objective of this study is to develop a low-cost photoanode by utilizing inexpensive precursors and processing techniques. Our preliminary studies indicate that pristine BiVO_4_ has a low photocurrent density. As a result, doping techniques were used to increase the photocurrent density and Mo will be used as a dopant. The direct solution synthesis of a Mo-BiVO_4_ is a difficult task. We describe a straightforward one-pot method for preparing bismuth-based precursors for photoelectrode materials. This contrasts with what has previously been reported in the literature regarding the use of an organic capping agent in the fabrication of ultrafine magnetic nanoparticles.^36^ The synthesis of Mo-doped BiVO_4_ is illustrated in Scheme 1A using a non-aqueous material processing route. Metal nitrate salts are reacted with oleic acid and oleyl amine to form a viscous mass, which is then reacted with an acetylacetonate complex of vanadyl and molybdenyl ions. Following tetrahydrofuran treatment, it forms a dispersion solution that when coated with FTO (fluorine-doped tin oxide) and thermally treated forms a Mo-BiVO_4_ thin film. Its chemistry can be explained by the proposed mechanism depicted in Scheme 1B.

Due to the hydrophobic nature of fatty acid alkyl chains, the thermal decomposition of nitrate salt in the presence of oleyl amine produces stoichiometric bismuth oxide seed particles. Oleic acid is also used in the reaction medium as a capping agent.^37^ As previously described,^37^ the reaction produces NO_2_ and oxygen gases as by-products. Following nanoparticle formation, bismuth oxide forms a weak hydrogen bond with the acetylacetonate ligands of both vanadyl and molybdenyl ions.^38^ In the THF dispersion, the unreacted vanadyl acac and molybdenyl acac remain as such. THF is added to the reaction mixture to disperse the complex formed due to its viscous nature. The dispersion was then used to fabricate bismuth vanadate films *via* dip coating and isothermal thermal treatment at temperatures ranging from 450 °C to 550 °C. The thermal treatment produced stoichiometric BiO_3/2_, V_2_O_3_, and MoO_*x*_ particles, which were then fused using a solid-state high-temperature chemistry approach to form Mo-doped BiVO_4_ particles, as depicted in the mechanism. MoO_*x*_ particles can also be formed during the thermal decomposition of molybdenyl acetylacetonate on solid surfaces.^39^

**Fig. 1:**
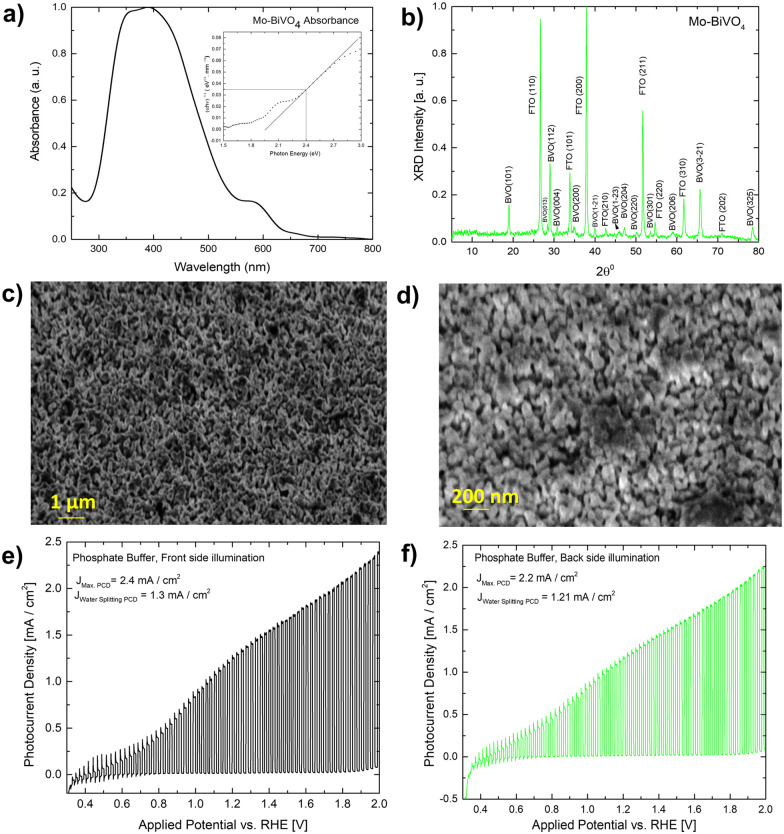
(A) UV-Vis absorbance of Mo-BiVO_4_ photoelectrode (B) thin-film X-ray diffractogram of Mo-BiVO_4_ photoelectrode; (C and D) shows both low and high-resolution FESEM imaging of the Mo-BiVO_4_ photoelectrode; (E) photocurrent densities of Mo-BiVO_4_ photoelectrode in phosphate buffer electrolyte (0.1 M, pH = 7) during front side and (F) back side illumination.

A UV-Vis absorbance study is used to characterize the obtained Mo-BiVO_4_ photoelectrode. As shown in Fig. 1A, the photoelectrode exhibits two absorption band maxima of 400 and 580 nm, which correspond to the previously reported magnitudes for Mo-BiVO_4_ photoelectrodes.^40^ The bandgap energy is determined using the Tauc methodology for indirect bandgap calculation.^41^ The bandgap value obtained is 2.4 eV, which confirms the electrode's ability to absorb visible light and agrees with previously published results.^40^ The absorbance of the Mo-doped BiVO_4_ photoelectrode changes as the layer thickness increases, followed by the transmittance pattern [Fig. S1, ESI†]. UV-Vis spectra are also generated for two additional parameters under investigation: the effect of doping concentration and the annealing temperature is as shown in Fig. S1 (ESI†). The annealing temperature does not affect the size of the nanoparticles generated because there is no evidence that the energy band gap value of the Mo doped BiVO_4_ n-type semiconductor photoanode changes. In addition, this study's findings are in line with previous research.^42^ Increased heat treatment temperature and longer residence time both cause a maximum shift towards longer wave lengths, which signifies a red shift phenomenon for particle size [Fig. S1, ESI†]. N_2_ treatment of BiVO_4_ photoanodes has yielded similar results, with the shift possibly being caused by formation of interband states because of the filling of the oxygen defect by nitrogen.^40^ This additional peak is the result of a doping-induced band gap transition, as observed in prior studies of Cr^6+^-doped BiVO_4_, as depicted in the figure below.^1^ In our investigation, Cr^6+^ is substituted for Mo^6+^. The electronic transition from the valence band to the acceptor level of dopant's empty 3d orbitals governs the new absorption behaviour.^43^ Following that, an X-ray diffractogram was taken to confirm the monoclinic clinobisvanite or scheelite type of bismuth vanadate [Fig. 1B] and it is noticed that the corresponding Bragg planes closely align the calculated clinobisvanite phase pattern.^44^ The detailed structural properties of each optimized component are shown in Fig. S1 [see the ESI†]. A significant finding is that the sample with the highest photocurrent density exhibits a dramatic increase in the Bragg peak diffraction intensity when compared to the corresponding planes of BiVO_4_(101)/(112). While the FTO(110) diffraction intensity remains constant here, this indicates that the thickness of the BiVO_4_ film has no effect as described in the ESI.† Additionally, to confirm the effect of molybdenum doping on the crystallographic structure of bismuth vanadate, a comparative XRD analysis between doped and undoped samples is performed [Fig. S3, ESI†]. It is evident here that the Bragg peak obtained at 18.6° Bragg angle splits into two distinct peaks for undoped bismuth vanadate, whereas this peak appears as a single peak for molybdenum doped bismuth vanadate. From this, it is demonstrated that doping with Mo has a significant effect on the crystallographic properties. The photoelectrode morphology is studied using FE-SEM (field emission scanning electron microscopy). Fig. 1C and D illustrate the FE-SEM imaging of the photoelectrode. When the temperature is increased from 472 to 550 °C, we observe well-distributed particles with strong inter-particle necking. The morphology is consistent with a previously published report on the morphology of bismuth vanadate photoanodes.^17^ Close examination of the high-resolution SEM imaging reveals that the particles are between 80 and 200 nm in size. Additionally, the particles are deposited in such a way that adequate porosity is maintained.

Fig. S8 (ESI†) illustrates the photocurrent densities of photoanodes optimized for various heat treatment temperatures. Before measuring the photocurrent density under chopped light conditions, each sample is subjected to linear sweep voltammetry (LSV). The photocurrent density of sample S 109 (heated to 470–550 °C) [Fig. 1E and F] is 2.4 mA cm^−2^ when illuminated from the front side at 2 V *vs.* RHE (light exposed *via* standard aperture size, while the water splitting current density remains constant at 1.30 mA cm^−2^. The illumination from the back side results in a photocurrent density of 2.2 mA cm^−2^. At the same applied potential, the total photocurrent density increases to 3.5 mA cm^−2^ with full aperture in front light illumination and 3 mA cm^−2^ under back side illumination [Fig. S8E, ESI†]. The photocurrent density observed is comparable to that of textured Mo-BiVO_4_ previously reported.^17^ When the applied potential is low, as evidenced by the spike formation, significant recombination losses occur in comparison to when the applied potential is high. BiVO_4_ exhibits significant recombination losses, which can be minimized in a variety of ways. For example, prior work demonstrated that recombination losses in the BiVO_4_-based water photooxidation process can be overcome using overlayer and underlayer coated materials that alter the semiconductor surface physics slightly. In this instance, the process has been explained using the hole mirroring phenomenon. SnO_2_ acts as a hole mirror underlayer by preventing hole migration into the back contact.^45^ To reduce efficiency losses due to excitonic recombination at defect sites, we'll use biomass as a hole acceptor. Section 2.3 will go into additional details about this.

### TEM (transmission electron microscopy) crystallographic and composition characteristics studies of Mo-BiVO_4_ photoelectrode and beam exposure studies to determine the effect on lattice fringe evolution

**Fig. 2:**
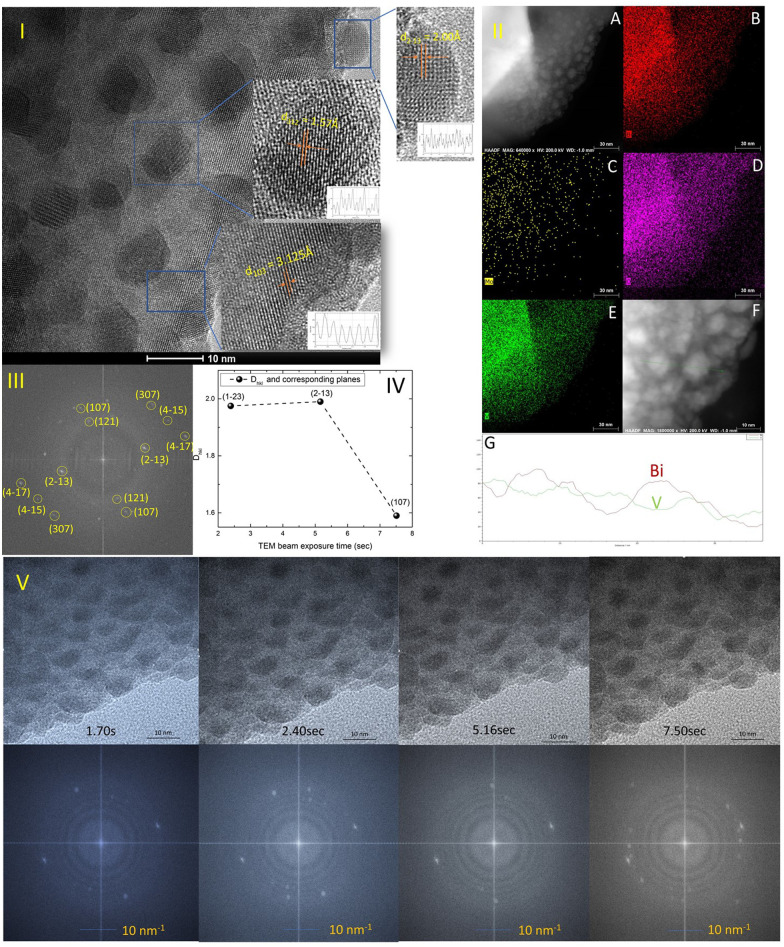
(I) The TEM image of Mo-BiVO_4_ nanocrystallites showing the lattice fringes with *d*-spacing well matched the clinobisvanite or scheelite type crystallographic structure. The zoom out section of the image on the right top reveal 2-D type crystallites as lattice fringes directed in both *X*–*Y*-axes direction; (II) (A) HAADF STEM imaging of Mo-BiVO_4_ nanocrystallites show the elemental compositions of Bi (B), Mo (C), O (D) and V (E); a line-scan analysis (G) shows the distribution of Bi/V ratio on a single nanocrystallite (F); (III) the FFT converted TEM images shows the electron diffraction spots match well with the observed X-ray Bragg diffraction pattern. (IV) The change in the *d*-spacing value of lattice fringes against beam exposure time demonstrates the different crystallographically oriented electron diffraction planes, (V) TEM snapshot of the beam (upper section) exposed nanocrystallites over a few seconds shows the dynamic evolution of crystallite lattice fringes. The below section shows the FFT conversion of images to validate the variation of the electron diffraction pattern.

The TEM study of the Mo-doped BiVO_4_ photoelectrode crystallites is being conducted to gain a better understanding of their crystallographic properties. The TEM image obtained [Fig. 2(I)] at a resolution of 10 nm reveals lattice fringes with a *d*-spacing value that is very similar to that obtained using XRD Bragg planes. The topmost zoom out crystallite fringes exhibit a two-dimensional pattern, and upon TEM beam exposure, new nano crystallites are formed. The *d*-spacing value is calculated using the Image J line scan methodology. To confirm the presence of molybdenum in the bismuth vanadate photoelectrode, EDX mapping was performed using HAADF STEM imaging, as illustrated in Fig. 2(II)-A. The mapping patterns shown in Fig. 2(II)B–E correspond to the Bi, V, and O elements. Molybdenum is also present in the Mo doped BiVO_4_ lattice, along with bismuth and vanadium, as revealed by the mapping analysis. Unlike this study, a TEM line scan analysis of newly formed crystallites [Fig. 2(II)F and G] revealed a consistent change in the Bi/V ratio. From the bulk to the surface, a line scan is performed across 40 nm of samples. As will be discussed below, it is obvious that the Bi/V ratio changes following the RBS finding. From this, the beam-exposed nano crystallites are composed of the BiVO_4_ phase, as confirmed by the TEM data's *d*-spacing value for crystallographic planes. To gain a better understanding of the crystalline lattice fringe nature, a static fast Fourier transformation of the TEM image is performed as illustrated in Fig. 2(III). The electron diffraction spot here closely matches the Miller indices of the BiVO_4_ crystal lattice as determined by phase identification of the corresponding XRD data. The effect of TEM beam exposure (200 kV) on lattice fringes is investigated by exposing crystallites for 12 seconds. From the cluster overlap of TEM images and their conversion to a video file obtained [the video file is attached in the ESI†], it is clear that the dynamic evolution of the nanocrystallite crystal fringes during the electron beam irradiation progresses like a recent finding of structural dynamics of redox metal catalysts using operando TEM.^46^ As illustrated in Fig. 2(V), various snapshots of the referred TEM imaging of lattice fringes have been taken. FFT conversion of TEM images taken at various beam exposure times demonstrates the variation in the electron diffraction spot associated with various Miller indices. The same is plotted against the TEM beam exposure time, revealing distinct crystallographically oriented electron diffraction planes [Fig. 2(IV)]. Continuous beam exposure for a few seconds is believed to form an additional layer of nano crystallites on the surface of the nanoparticle, as the majority of new nanocrystallite with differently evolved fringes formed on the sample's edge. Additionally, the video depicts a dynamic crystal lattice variation on the molybdenum doped BiVO_4_ particles' very top surface. This is the first time that this type of dynamic rearrangement of crystallite lattice fringes has been observed for Mo-BiVO_4_.

### Solar glycerol oxidation to produce hydrogen with the Mo-BiVO_4_ photoanode

Recently, photoelectrochemical biomass oxidation or solar biomass splitting on semiconductor surfaces has been proposed as another method for decreasing charge-carrier recombination and lowering the overpotential, which is analogous to the sulphite oxidation process described in almost all of the work discussed above.^7,17,38,40^ Sodium sulphite was used as a hole scavenger in this case. However, solar biomass splitting has an advantage in terms of organics conversion and hydrogen production.

To increase the photocurrent density of bare Mo doped BiVO_4_ photoelectrodes, we exploit a reaction on their surface that requires less thermodynamic energy input, such as photoelectrochemical glycerol oxidation. This reaction requires less energy than water splitting since it is a one-step electron transfer reaction, whereas water splitting is a multi-step reaction.^47^ We can increase both the efficiency and the amount of hydrogen produced by this reaction on the Mo-BiVO_4_ surface. Notably, hydrogen is generated *via* the light-mediated oxidation of the glycerol molecule, as illustrated in chemical eqn (1).
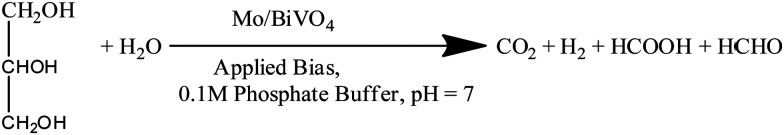


**Scheme 2:**
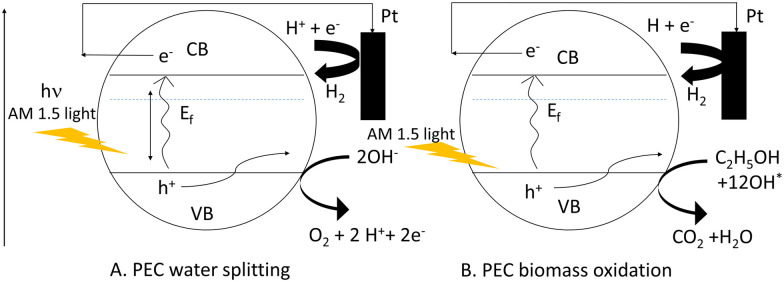
Pictorial depiction of reduction of recombination loss seen in PEC water splitting (A) by PEC based biomass oxidation chemistry (B).

Considering this, ethanol and methanol have already been used to minimize photoelectrode recombination losses when used as a hole scavenger. The bandgap energy level can be used to illustrate the comparison of PEC-based water splitting and PEC-based biomass oxidation processes in terms of recombination losses as shown in Scheme 2. In this case, we considered two Mo-BiVO_4_ particles denoted by the letters A and B. Here, we can easily see that while the PEC-based water splitting process may result in increased recombination losses, the PEC-based biomass oxidation process avoids this situation because the biomass acts as an electron donor or hole scavenger. In the latter case, fewer holes are consumed by biomass oxidation, increasing overall photocurrent density.

**Fig. 3:**
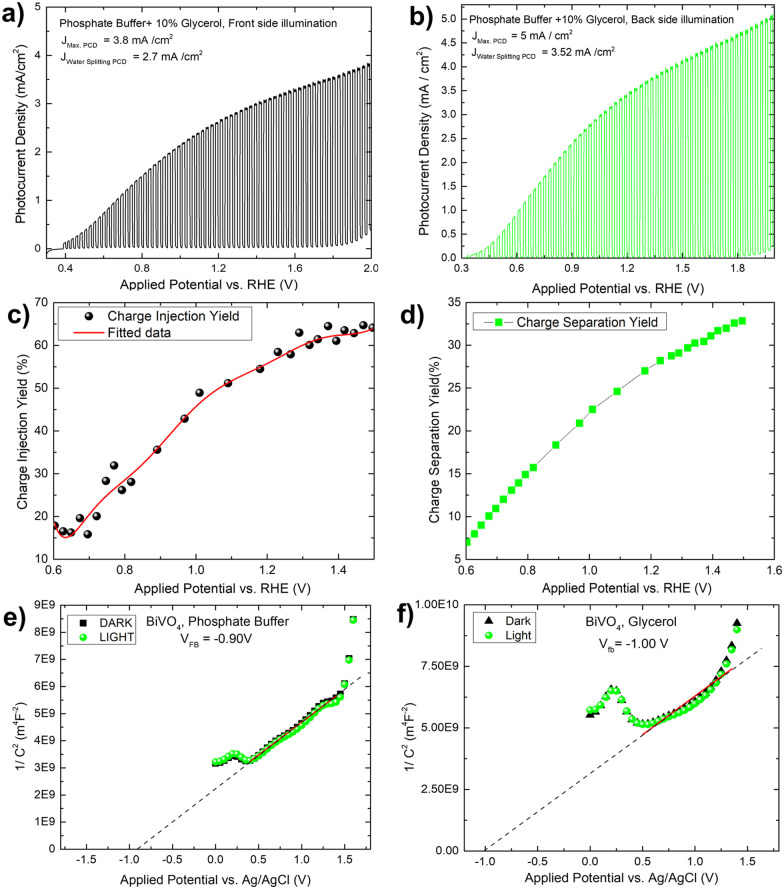
Transient photocurrent densities of Mo-BiVO_4_ photoelectrode in the phosphate buffer electrolyte (0.1 M, pH = 7) plus 10% glycerol during front side A and B back side illumination; C and D shows charge injection and separation yield calculation for the Mo-BiVO_4_ photoelectrode by considering glycerol as hole scavenger instead of H_2_O_2_; (E and F) flat band evaluation using Mott–Schottky plot for the Mo-BiVO_4_ photoelectrode in phosphate buffer and glycerol under dark and light conditions.

To verify the solar biomass oxidation process, we first determined the photocurrent density of a Mo-BiVO_4_ photoelectrode when illuminated from the front side in the presence of glycerol. When glycerol (10 ml) is added to the 0.01 M phosphate buffer electrolyte, the photocurrent density of the Mo-BiVO_4_ photoelectrode rapidly increases from 0.3 V to 2.0 V vs RHE with a magnitude of 3.8 mA cm^−2^, as shown in Fig. 3A. The current density of water splitting is 2.7 mA cm^−2^ at 1.2 V. Back side illumination [Fig. 3B] begins at the same potential as front side illumination and gradually increases to a saturated current density of 5 mA cm^−2^. The current density of the water splitting is greater than that of the front side illumination and is set to 3.5 mA cm^−2^. The charge separation and injection yield of the Mo-BiVO_4_ photoelectrode [Fig. 3C and D] was then calculated considering glycerol acts similarly to hydrogen peroxide as a hole scavenger. Charge separation and injection yield are calculated in this section using the protocol described previously,^48^ and the light-harvesting efficiency [Fig. S16, ESI†] is calculated before finding out the absorbed and maximum photocurrent densities respectively.^7^ Once we get the value of *J*_abs_ we can first calculate the separation yield by adopting the following formula as described in ref. 48. We have not obtained though exactly 100% charge injection yield, which is obtained when glycerol is highly concentrated as described in ref. 32. At 1.4 V, the charge injection yield increases to 65% and the charge separation yield increases to 32%. The Mott–Schottky plot of the Mo-BiVO_4_ photoelectrode in glycerol and phosphate buffer is shown in Fig. 3E and F and was calculated according to the protocol described elsewhere.^49^ The flat band potential is found to remain constant regardless of whether glycerol or water is oxidized photoelectrochemically. Thus, photooxidation of glycerol does not affect the band bending of the photoelectrode at the semiconductor–electrolyte interface.

### Photocharging of the Mo-BiVO_4_ photoelectrode by glycerol oxidation

**Fig. 4:**
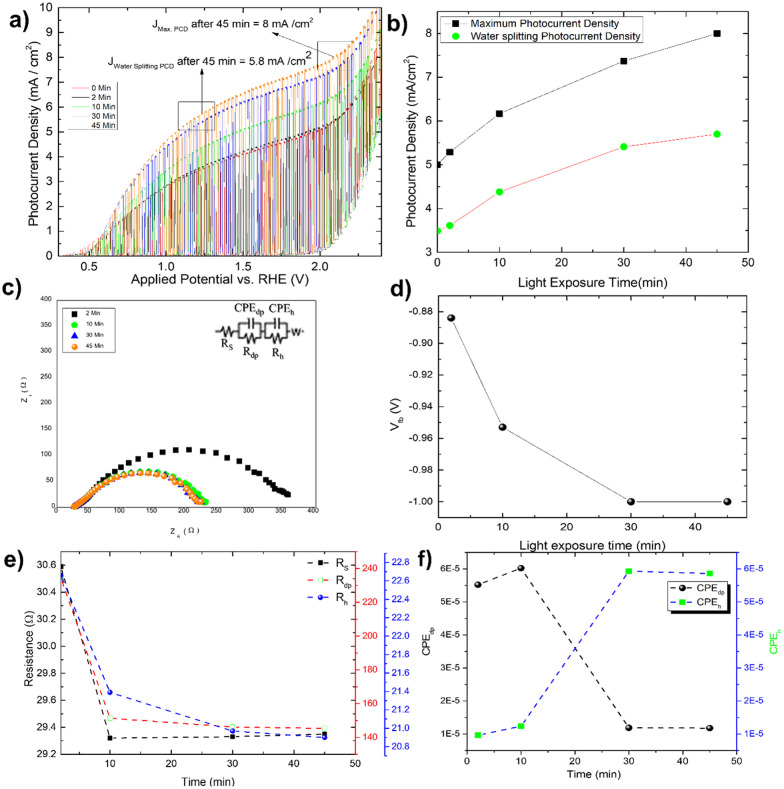
(A) Photocharging effect shown by Mo-BiVO_4_ in glycerol followed by an increase in light exposure time; (B) variation of both maximum and water splitting current density for light exposure time; (C) the Nyquist plot showing the variation of impedance pattern and (D) variation of flat band potential (*V*_fb_) with light exposure time; (E) variation of charge transfer resistance across solid (*R*_s_), depletion layer (*R*_dp_) and Helmholtz layer (*R*_h_) following photo charging time; (F) variation of capacitance element across depletion layer (CPE_dp_) and Helmholtz layer (CPE_h_) to photo charging time.

By measuring the photocurrent density in the presence of glycerol, the photo charging effect of Mo-BiVO_4_ is investigated. It should be noted that the photo charging effect^24–26,50^ of BiVO_4_ is a well-known phenomenon and is mostly observed for basic electrolytes. The effect on the photocurrent density saturation limit is shown here. The experiment is carried out by exposing the electrode for 2 minutes to 45 minutes under open circuit condition (OCP), and as the time passed, the photocurrent density increased and reached a *J*_max_ of 8 mA cm^−2^ at 2. 0V *vs.* RHE [Fig. 4A]. After 45 minutes of photo charging, the maximum water splitting current density *J*_water splitting_ at 1.23 V reached 5.8 mA cm^−2^. Fig. 4B depicts the variation in each case. We tried this for 45 minutes until the photocurrent density saturated. This is the first observation and effect of its kind for photoelectrochemical glycerol oxidation and very high photocurrent density in comparison to already published work as depicted in Table 1. For the BiVO_4_/Hematite heterojunction system, a comparable photocurrent density is found.^22^ In both instances, the photocurrent density exceeds the band gap absorption limit because of charge transfer activity, particularly in photocharging effect.

**Table 1:** Photocurrent density comparison 1.2V *vs.* RHE with the already published work

	Pristine (photocurrent density (mA cm^−2^)	Co-catalysts (photocurrent density (mA cm^−2^)	Glycerol (photocurrent density (mA cm^−2^)	Applied bias *vs.* RHE (V)	Ref.	Remark
W-BiVO_4_	2.3	3.3	4.2	1.2	*J. Mater. Chem. A*, 2021, **9**, 6252–6260.^35^	Electrolyte = KBi and Na_2_SO_4_ besides glycerol and co-catalysts
BiVO_4_	1.2	NA	4	1.2	*Nat. Commun.*, 2019, **10**, 1779.^33^	Electrolyte = Na_2_SO_4_ besides glycerol
BiVO_4_	1.5	NA	1.5	1.2	*Electrochim. Acta*, 2019, **322**, 134725.^32^	Electrolyte = Na_2_SO_4_ besides glycerol
BiVO_4_	1.5	NA	1.5	1.2	*App. Catal. B: Environ.*, 2020, **278**, 119303.^34^	Electrolyte = NaBi besides glycerol
Fe_2_O_3_/BiVO_4_	4	NA	NA	1.2	*Nat. Commun.*, 2016, **7**. https://doi.org/10.1038/ncomms13380.^22^	Electrolyte = KPi; Maximum photo current density = 8 mA cm^−2^
WO_3_/W:Mo-BiVO_4_	4	5	NA	1.2	*Nat. Commun.*, 2015, **5**, 4775.	Electrolyte = K_2_SO_4_; with Na_2_SO_3_ as hole scavenger
W-BiVO_4_	1	3	NA	1.2	*Nat. Commun.*, 2013, **4**, 2195.^21^	Electrolyte = Phosphate buffer
BiVO_4_	4	5	NA	1.2	*Nat. Chem.*, 2015, **7**, 328.^27^	Electrolyte = Phosphate buffer; nitrogen treatment
Mo-BiVO_4_	2	NA	3.5	1.2	This work	Electrolyte = Phosphate buffer besides glycerol; no co-catalysts; pH = 7
Mo-BiVO_4_	NA	NA	5.8	1.2	This work	After photocharging phenomenon, Maximum Photocharging current density = 8 mA cm^−2^ in the presence of glycerol

The increased band bending on the photoelectrode's surface is thought to be responsible for the photocharging effect, which improves charge separation and suppresses charge recombination. In a borate modified BiVO_4_ layer, ionic adsorption on the BiVO_4_ surface leads to the creation of heterojunction, as previously observed.^50^ Adsorption of glycerol on bismuth based electrocatalyst has recently been observed.^51^ It is expected that glycerol will produce an absorbate layer on the bismuth vanadate surface, hence improving band bending and increasing photocurrent density, because of photocharging effects. Another intriguing observation is the change in the impedance behaviour of the photoelectrode after each minute of photo charging. Analysing the electrochemical impedance Nyquist plot [Fig. 4C], it is found that the smallest semicircle obtained from 5 min to 45 min photo charging indicates less charge transfer resistance and thus an increase in the photocurrent density. However, the semicircle shifted from the origin is likely due to the presence of glycerol which increases the solution resistance of the overall system. The addition of Warburg resistance in the Randles circuit as shown in the inset of Fig. 4C point towards kinetic and diffusion-controlled process. In this case, the flat band potential [Fig. 4D] ranges from −0.88 V to −1.0 V, reaching the previously mentioned standard *V*_fb_ for Mo-BiVO_4_. While fitting the EIS spectra with the model circuit and analysing the data, the different types of resistances for various units such as depletion and Helmholtz layer are revealed. In all cases, it is found that resistance decreases with increasing photo charging time [Fig. 4E]. Next, we found that the capacitance evaluated by the constant phase element^49^ reveals that the capacitance decreases at the depletion layer as the photo chargic time increases, whereas it increases for the Helmholtz layer in the opposite direction. The photo charging effect is valid here due to a change in the capacitance value of the Helmholtz layer. This type of surface capacitive layer is found to capture the holes generated and thus decreases the recombination losses.^27^

### IPCE, rate of hydrogen evolution, faradaic efficiency and Photostability studies of Mo-BiVO_4_ photoanode in the presence and absence of glycerol

**Fig. 5:**
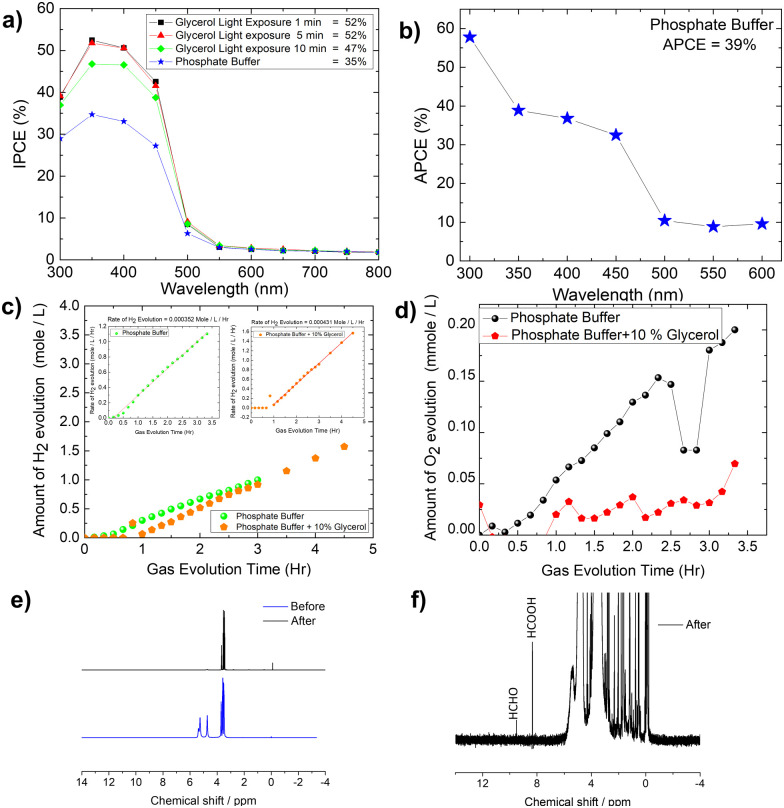
(A) Effect of photo charging time on the IPCE (incident photon to current conversion efficiency) of Mo-BiVO_4_; (B) APCE (absorbed photon to current conversion) efficiency of Mo-BiVO_4_ in phosphate buffer; (C) operando GC quantification of hydrogen gas evolution from both phosphate buffer and with glycerol, inset: rate of hydrogen evolution calculation from the slope of GC data for both phosphate buffer and glycerol; (D) quantification of O_2_ evolution in both phosphate buffer and glycerol; (E and F) ^1^H NMR spectra of the electrolyte collected after photoelectrochemical glycerol oxidation.

**Fig. 6:**
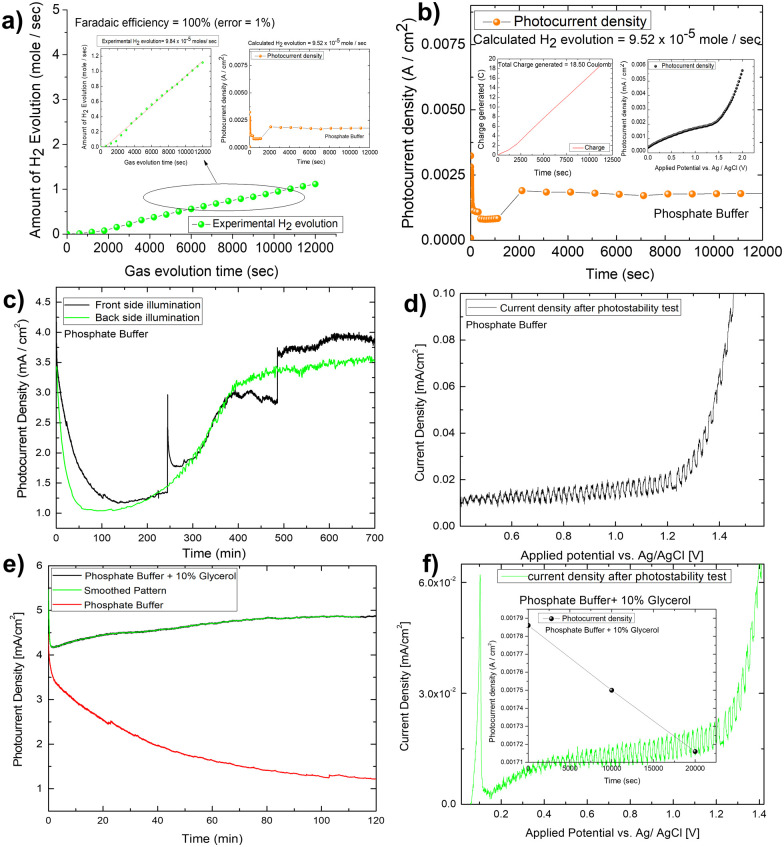
(A) Calculation of faradaic efficiency for hydrogen production using Mo-BiVO_4_ in phosphate buffer; (B) photocurrent density of the photoelectrode during hydrogen evolution reaction and calculated hydrogen evolution rate; (C) long term chronoamperometric investigation of the photoelectrode for around 11 h in both front and back side illumination; (D) photocurrent density of photoelectrode after long term stability test; (E) long term chronoamperometric stability study of Mo-BiVO_4_ in glycerol for 2 hours and (F) the validation of photoactive nature of the electrode after long term stability test.

The IPCE (incident photon to current conversion efficiency) of the Mo-BiVO_4_ photoelectrode was then measured in both pristine and photocharging conditions. In this study, we found that the IPCE of the electrode in PBS buffer is 35%, but increases to 52% after glycerol oxidation. Fig. 5A shows that increasing the photocharging time reduces it to 47%. In a wavelength range of 350–450 nm, the electrode's absorbed photon to current conversion efficiency (APCE) is 39%. Quantitative gas evolution study was then carried out using the operando GC (gas chromatography) method. In Fig. 5C and D, the evolved hydrogen and oxygen gas concentrations are plotted separately for phosphate buffer and glycerol. The trend of hydrogen evolution in both cases is evident here, though, after 3 hours, glycerol oxidation leads to a greater amount of hydrogen evolution. The inability to measure the hydrogen concentration in phosphate buffer after 3 hours is due to the electrode's unstable nature. BiVO_4_ photoelectrodes are highly photo unstable. During glycerol oxidation, a parasitic oxidation reaction aids in the maintenance of photocurrent density for a longer period. The following section delves deeper into it. The oxygen evolution concentration for glycerol oxidation, on the other hand is low, whereas it is higher for phosphate buffer electrolytes because the water-splitting reaction results in a 2 : 1 nature of gas evolution. The low oxygen concentration is compensated for by the formation of organics such as formic acid and formaldehyde, as measured by liquid ^13^C NMR (Fig. 5E and F). We did not put the error bar here as each calculated rate of gas evolution is the measurement of 3–5 reading of the GC spectrum. We have taken the average PPM to calculate the rate of hydrogen evolution. It is the exact value that we have obtained after translating the raw data. We have made a comparison in the rate of oxygen evolution of both water oxidation and glycerol oxidation as shown in Fig. 5D as an alternative to the isotope labelling experiment. The reason behind this experiment is to observe the quantity of oxygen evolved as PEC glycerol oxidation only evolves organics and hydrogen according to the following equation. Next, we calculated the rate of hydrogen evolution [inset of Fig. 5C] for both electrolytes, and the results are 0.000353 mol l^−1^ h^−1^ and 0.000431 mol l^−1^ h^−1^, respectively. Based on these values, calculation of half-cell STH efficiency by subtracting off the hydrogen evolution electrode potential (0 V) from that of thermodynamic water splitting potential for the oxygen evolution reaction (1.23 V) with the help of equation described elsewhere.^8^ It is found to be 5.5% for pure water splitting. Following that, we calculated the photoelectrode's faradaic efficiency for hydrogen formation. As shown in Fig. 6A, a normal phosphate buffer has 100 per cent efficiency in the formation of hydrogen gas. In this case, the experimental hydrogen evolution concentration is 9.84 × 10^−5^ mole s^−1^. The theoretical hydrogen evolution concentration, on the other hand, is calculated from the integration of the chronoamperometry data, as shown in Fig. 6B. The integrated current density is 18.50 Coulomb, resulting in a hydrogen gas concentration of 9.52 × 10^−5^ mole s^−1^. Chronoamperometry experiments are used to investigate the stability of the best-optimized films. The chronoamperometric (CA) experiment is carried out over 12 hours with a bias of 1.8 V in phosphate buffer. The photocurrent density of the electrode decreases from 3.5 mA cm^−2^ to 1.2 mA cm^−2^ after 100 minutes of operation under front side illumination during the CA scan [Fig. 6C].

Photocurrent density degrades faster on the backside illumination than on the front side illumination after 50 minutes. The current density for back side and front side illumination has significantly improved after 200 minutes of operation. This high onset in current density, however, is primarily due to the faradaic oxidation process, as most of the photoactive materials on the electrode surface corrode. This is supported by the lower spike response during chopped photocurrent density measurement [Fig. 6D]. The gas bubble evolution causes the spike formation for front-side illumination in the CA investigation. This discussion concludes that the prepared photoelectrode is not stable. However, to see if glycerol oxidation aids photoelectrode stability, we conducted a 2 hour long-term study in the glycerol mixed phosphate buffer [Fig. 6E]. In this case, the current density increases for the first two hours in a pattern like that of the protein functionalized hematite film discussed elsewhere.^52^ This increase in current density is required for a sustainable operation, but the light chopping current does not show any significant improvement in photoelectrode stability after the LCE (long term electrochemical operation) [Fig. 6F].

Though the doped BiVO_4_ photoanode performs well, it has a significant drawback in terms of photodecomposition, limiting its use as an efficient material for photoelectrochemical hydrogen production. For example, a recent study suggested that by utilizing a V^5+^ ion saturated electrolyte, the electrolyte composition might be tailored to decrease the photo corrosion response. Photo corrosion occurs when V^5+^ is removed from the BiVO_4_ lattice. Additionally, it increases the kinetics of OER by integrating it with the co-catalyst layer.^53^ Another work used *in situ* AFM to develop a precise mechanistic explanation for the BiVO_4_ electrode's photostability. The build-up of photoexcited charge carriers on the surface destabilizes the lattice, resulting in kinetic hindrance and the creation of a chemically stable, self-passivated surface.^54^ By and large, the Ni(OH)_2_ co-catalyst layer absorbs excess holes on the BiVO_4_ layer, oxidizing Ni^2+^ to Ni^3+^ and generating NiOOH. Followed by this, the small presence of photoactivity in the BiVO_4_ after glycerol oxidation is due to the removal of photoexcited charge carriers which helps in the photodecomposition reaction. Normal photoelectrochemical water splitting on a bismuth vanadate electrode results in photodisintegration of the electrode. However, the solar biomass splitting induced photo charging effect contributes to extending the stability period to some extent with the help of glycerol as a hole scavenger. When compared to conventional PEC water splitting, the efficiency of solar to hydrogen conversion increases. If biomass feedstock is available, solar biomass oxidation may be a viable alternative to producing hydrogen fuel due to its increased efficiency.

### Investigation of the post photoelectrochemically processed samples in terms of optical, structural, and compositional properties followed by morphological properties

#### Optical property studies

**Fig. 7:**
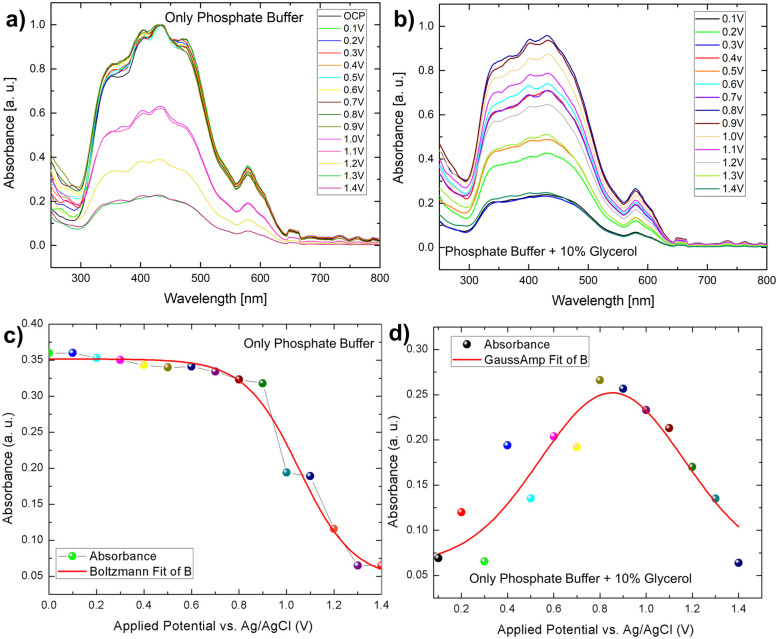
(A and B) *In situ* spectroelectrochemical understanding of Mo-BiVO_4_ photoelectrode in the presence and absence of glycerol; (C and D) variation of absorbance pattern to applied potential in phosphate buffer and glycerol.

The optical properties of the Mo-BiVO_4_ photoelectrodes are monitored in the presence and absence of glycerol using an *in situ* UV-Vis spectrometer [Fig. S16, ESI†]. The experimental details are discussed in the section Materials and methods. The UV-Vis spectrum of the photoelectrodes in the spectro electrochemistry mode reveals interesting observations for the photoelectrode's light absorption properties concerning applied bias. It should be noted that the experiment is conducted entirely in the dark. This means that the standard AM1.5 solar lamp is not used in this case. It will lead to a better understanding of the effect of the electrolyte and biomass distribution environments on light absorption at the semiconductor–electrolyte interface. We found that the absorbance of a Mo-BiVO_4_ photoelectrode at 560 nm varies slightly until the applied bias reaches 0.9 V. Following that, from 1.0 V to 1.4 V, the absorbance follows a decay pattern in the form of a Boltzmann distribution obtained by the analytical fitting of the scattered data (Fig. 7a–c). This observation is due to the photo corrosion phenomenon, which occurs at a higher bias due to dark faradaic oxidative processes. We observed that the absorbance varies only in a decayed pattern across the entire potential window. This demonstrates that the parent electrode Mo-BiVO_4_ is photodegradable in the presence of phosphate buffer.

However, in the presence of glycerol, the variation of light absorbance concerning bias behaved somewhat differently than in the presence of only phosphate buffer electrodes. In this case, we observed that the absorbance intensity follows a gaussian distribution, which was not observed in the case of phosphate buffer (Fig. 7b–d). The distribution of biomass molecules at the semiconductor–electrolyte interface can be used to explain this behaviour change. The following discussion can be drawn from the pattern of scattered light absorbance magnitude for applied bias. At 0.1 V, the light absorbance remains constant because most of the light active part of bismuth vanadate is covered by a layer of glycerol molecules. At 0.2 V, we saw a slight increase in absorbance, which then decreased at 0.3 V. This is qualitatively understood as the glycerol molecule layer oxidizes, allowing light to be absorbed by the photoactive layer while photocurrent increases because of photoelectrochemical water oxidation and the glycerol scavenging action of the light generated holes. The chronoamperometry mode is used for this operando experiment. That is, we run the current vs time experiment at a fixed voltage to prepare the electrode to see the after effect of each voltage window. The time between measurements is nearly 30 seconds. During these 30 seconds, glycerol molecules filled the gap left by an already oxidized glycerol molecule at the semiconductor electrolyte interface, preventing light from reaching the photoactive layer and decreasing absorbance.

A similar pattern has evolved in the next voltage window, 0.4–0.5 V, with a slight increase in the absorbance, compared to 0.2 V. After that, absorbance increases continuously in the same pattern until it reaches a saturation maximum at 0.8 V, then decreases in magnitude and reaches a minimum in the voltage window of 1–1.4 V. It is worth noting that after this applied voltage of 1.8 V, the electrode exhibits chopped light activity and is thus light stable. We used a fixed potential of 1.8 V based on the photoelectrode's stability study. As previously stated, the photocurrent density increases over time in the presence of glycerol as well as in the absence of glycerol and follows the same pattern. Despite this, it can be concluded that glycerol, in conjunction with the continuous light exposure action, increases the charge transfer action of the photoelectrode, which essentially has some passivating action. Furthermore, the operando spectro electrochemical study indicates that the device operation should be performed at 0.8 V to reap the greatest benefits of light absorption. Following this photocurrent competition, the dark faradaic oxidation process occurs. As a result, the observed photocurrent density is a combined effect of dark water oxidation and photoelectrochemical glycerol oxidation (Fig. 6).

##### Correlation of IPCE and photostability using spectro electrochemistry

As discussed in the IPCE section, the electrode only works in the visible light region 350 nm–500 nm. The operando absorption spectrum discussed above also forms exactly in the same range with the maximum absorbance at 0.8 V implying maximum IPCE in glycerol mediated oxidation. After this and continuous light exposure, the efficiency of light absorption decreases, but the continuous increase in current density indicates that light-mediated PEC oxidation is overcome by the faradaic oxidation process, providing a reliable understanding of the electrode stability. The higher efficiency of the electrode in the presence of glycerol leads us to believe that the electrode without glycerol will have a longer photo degradability time.

#### Structural and morphological properties studies

**Fig. 8:**
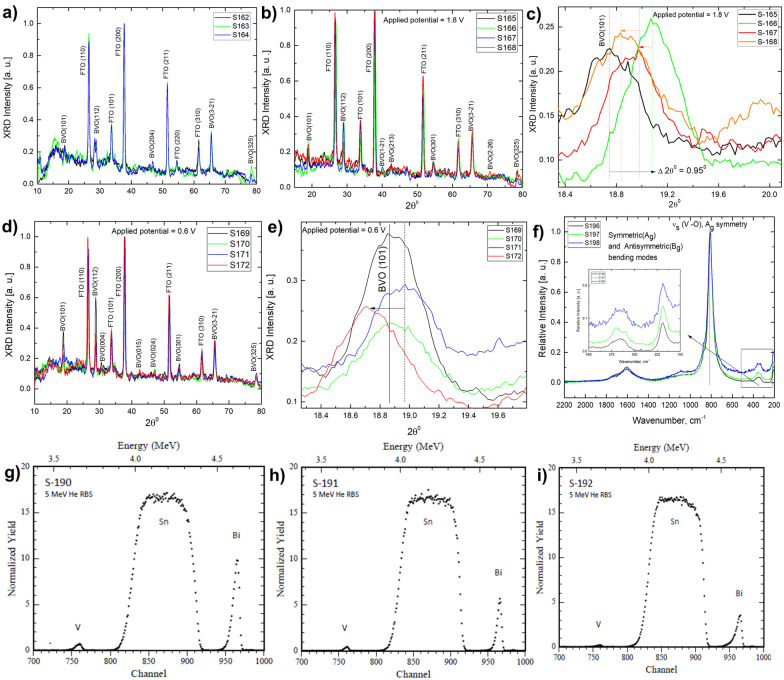
*Ex situ* X-ray diffraction of photoanode (A) after electrolyte treatment in only Phosphate buffer and glycerol plus phosphate buffer; (B) after running linear sweep voltammetry at 1.8 V in Phosphate buffer and glycerol under dark (S-165) and light (S-166) conditions; (C) expanded X-ray diffractograms showing (101) peak shifting; (D) after running linear sweep voltammetry at 0.6 V in Phosphate buffer and glycerol under dark (S-165) and light (S-166) conditions; (E) expanded X-ray diffractograms showing (101) peak shifting; (F) *ex situ* Raman spectral investigation of photoanode in electrolyte having only Phosphate buffer and glycerol plus phosphate buffer; (G) Rutherford Back Scattering (RBS) spectrum of pristine photoanode and in electrolyte having only Phosphate buffer and glycerol plus phosphate buffer.

Fig. 8(A–C) depicts X-ray diffractograms of post-electrochemically processed Mo-BiVO_4_ films (e) with different experimental conditions as shown in Table S5 of the ESI.† The XRD of Mo-BiVO_4_, in buffer and glycerol, followed by a long term chronoamperometric scan at 1.5 V under dark and light conditions in phosphate and glycerol, is considered here. Following that, the same set of samples is subjected to an *ex situ* XRD investigation while being chronoamperometrically scanned at the water-splitting voltage, which in this case is 0.6 V according to the Nernst equation. There have been no significant changes in the Bragg angle position in any of the cases studied. Only in the final set of samples does the peak (101) associated with the crystallographic structure of BiVO_4_ shift to a lower Bragg angle. However, when compared to the JCPDS pattern for the clinobisvanite phase, it perfectly matches that of BiVO_4_. As a result, the electrochemical treatment does not affect the crystallographic structure. The Raman spectrum of pristine Mo doped BiVO_4_ (S-196; Table S9, ESI†) samples shows the usual symmetric (A_g_) and antisymmetric (B_g_) bending modes, with a slight increase in the intensity of V_s_ (V–O) stretching modes corresponding to A_g_ symmetry. Under electrochemical conditions, the PBS buffer (pH = 7) treatment increases the intensities of the A_g_ and B_g_ modes in a deconvoluted fashion into two distinguishable peaks while not affecting the V_s_ (V–O) stretching modes. There is a significant change in the atomic composition of Mo doped BiVO_4_ after the glycerol treatment [sample conditions shown in Table S8, ESI†], which is correlated with the RBS data [Fig. 8G–I]. We believe that the treatment stretches the chemical bonding of the BiVO_4_ photoanode crystalline lattice, leaving a more pronounced signature in terms of changes in the Bi/V ratio.

In addition, FESEM images of photoelectrochemically processed samples revealed various morphologies in the presence of phosphate buffer and glycerol plus phosphate buffer [Fig. S13, ESI†]. EDX mapping shows that the bulk atomic composition of Bi and V differs, while the oxygen concentrations for all samples used in the studies differ. It should be noted that controlling the atomic composition is extremely difficult. The elemental composition of FESEM-EDX [Fig. S13, ESI†] varies in the atomic percentage of different elements at different electrochemically conditioned samples in the presence of glycerol and buffer.

#### Compositional properties

To address this issue, we examined the bulk composition of the samples using the Rutherford backscattering technique, which typically shows a similar trend in the V/Bi ratio for pristine Mo-doped BiVO_4_, in the presence of PBS buffer but the ratio changes when in contact with glycerol mixed PBS electrolyte. RBS data [Fig. 8(G–I)] show that the concentration of vanadium is decreasing with respect to bismuth. This result is also evident in the increased photodecomposition of the BiVO_4_ photoanode with the consequent loss of V and Bi.^54^ Vanadium here is present as VO_4_^2−^ ions. The V/Bi ratio changes here, indicating that glycerol affects the final performance of the electrode through some structural re-arrangement. The long-term chronoamperometric study shows that glycerol oxidation promotes stability when compared to the absence of glycerol *via* a dark reaction with respect to the usual photodecomposition pathway.

**Fig. 9:**
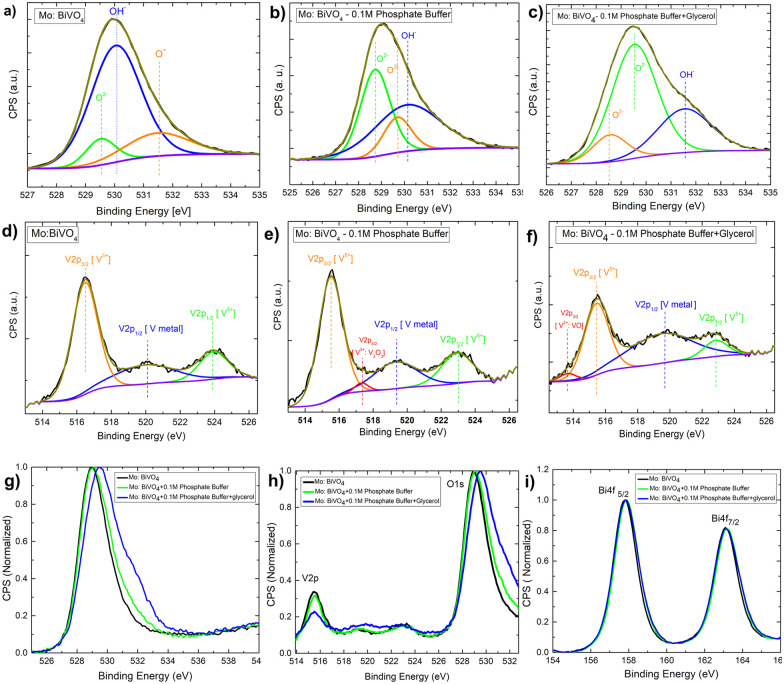
(A–C) O 1S core level XPS spectra of the pristine Mo-BiVO_4_ photoelectrode and in the electrolyte having only phosphate buffer and glycerol plus phosphate buffer; (D–F) V 2p core-level XPS spectra of the pristine Mo-BiVO_4_ photoelectrode and in the electrolyte having only phosphate buffer and glycerol plus phosphate buffer; (G–I) Bi 4f core-level XPS spectra of the pristine Mo-BiVO_4_ photoelectrode and in the electrolyte having only phosphate buffer and glycerol plus phosphate buffer.

Next, to understand the surface composition of the electrodes after electrochemical treatment, the XPS core level spectra were taken and thoroughly analysed [Fig. 9]. The Mo doped BiVO_4_ O1s core level XPS spectra reveal the presence of three chemical state signatures, O^2−^, OH^−^, and O^−^, in deconvoluted patterns at 529.5 eV, 530.1 eV, and 531.5 eV. The electrochemical treatment of Mo-doped BiVO_4_ with 0.1 M phosphate buffer resulted in the O^−^ peak shifting to the O^2−^ region and the OH^−^ peak shifting to the higher binding energy. Further treatment with glycerol during photoelectrochemical oxidation shifts the spectral weight of OH^−^ to the higher binding energy [Fig. 9(A–C)]. This indicates that the electrode has been oxidized with a higher charge transfer kinetics. The overall core level spectrum shows a positive shift in the direction of the higher binding energy, revealing that during photoelectrochemical glycerol oxidation, the electrode is oxidized, releasing electrons. In this case, the OH^−^ species can also be derived from the CH_2_OH linkage because of the photoelectrochemical breakdown of the glycerol molecule. We observed chemical states of vanadium with an extra shoulder peak between spin–orbit coupling peaks of V2P_3/2_ and V2P_1/2_ at 516.2 eV and 524 eV for V^5+^ in the V2P core level spectrum [Fig. 9(D–F)]. For V^2+^ metallic states, this extra peak is assigned to V2P_3/2_. Following electrochemical treatment with 0.1 M phosphate buffer, a new satellite peak at 517.2 eV is observed, which is assigned to metallic V from the V2P_1/2_ state. The formation of V_2_O_3_ results in the evolution of deconvoluted peaks at 517.4 eV, which is assigned to the V2P_1/2_ state of the V^3+^ ions. This peak disappears during the glycerol treatment, and a new peak appears at 513.6 eV. This peak corresponds to V2P_3/2_ states of V^2+^ ions formed during VO formation. While this shift is very slight, an *ex situ* study does not reveal the formation of such phases. It is also clear from the RBS study that the surface concentration of V is decreasing. The formation of vanadium based sub-phases helps in increasing the current density in dark mode for both phosphate buffer and glycerol based electrochemical reaction due to partial oxidation of V^2+^ and V^3+^ states into V^5+^ thereby preventing the complete photodecomposition. It is found that V^5+^ ions from crystalline lattice is responsible for decreasing photostability of the bismuth vanadate photoanode^53^ as mentioned above. In the case of the Bi4f scan [Fig. 9(G–I)], the spin–orbit coupling peaks remain the same in all cases. The presence of all elements is indicated by the survey spectrum. The carbon concentration is higher in the case of a 0.1 M phosphate and glycerol-containing film [Fig. S14, ESI†].

## Experimental

### Materials

Bi(NO_3_)_3_·5H_2_O, VO(acac)_2_, MoO_2_(acac)_2_, oleic acid, oleyl amine, and octadecyl amine were obtained from Sigma Aldrich in Switzerland. Dried ethanol and tetrahydrofuran are the solvents used for this purpose and are procured from Sigma Aldrich and Merck. All chemicals obtained are 99.9% pure and can be used without further purification. The glycerol and potassium phosphate (K_2_HPO_4_) used to make the phosphate buffer for electrochemical measurement is 99% pure and can be procured from Sigma Aldrich and Fischer in Switzerland. The water used to make the electrolyte has been double distilled.

#### Synthesis of a precursor for the Mo-doped BiVO_4_ film

BiVO_4_ thin-film precursors were synthesized using a non-aqueous surfactant-based processing route. In this case, Bi(NO_3_)_3_. 5H_2_O, VO(acac)_2_, and MoO_2_(acac)_2_ are combined in a 5 : 4 : 1 millimolar ratio in oleic acid, oleyl amine, and octadecyl amine mixture. The following is the detailed procedure: To begin with, 1 g octadecyl amine is mixed at room temperature with 30 ml of oleic acid and 20 ml of oleyl amine in a glass beaker and heated on a hot plate at 120 °C. The above mixture is then treated with a 2.5 g solution of bismuth nitrate pentahydrate [Bi(NO_3_)_3_·5H_2_O], and the reaction bath solution turns creamish in colour. The temperature of the reaction bath is gradually increased to 180 °C in the following step, while the solution becomes turbid. The inorganic and organometallic salt solution is prepared in 10 mL of absolute ethanol. The temperature is gradually increased to 200 °C with manual stirring until a dark green suspension is formed after adding 1 g of vanadyl acetylacetonate [VO(acac)_2_] solution (green color). Following that, as the temperature rises to 250 °C, a yellow-coloured suspension is formed. The reaction bath is then treated with 0.3 g of molybdenyl acetylacetonate [MoO_2_(acac)_2_], and the solution turns dark yellow green. Finally, the reaction temperature is maintained at 270 °C until a viscous mass is obtained.

#### Dip coating method for the synthesis of the Mo doped BiVO_4_ film

The viscous mass can dry overnight inside the fume hood. Following that, a suspension of the same is made in tetrahydrofuran (30 ml), which is then centrifuged at 7500 rpm for 6 minutes. It yields a precursor supernatant, which is used to create the Mo doped BiVO_4_ thin film. Manual dip coating is used, in which a FTO glass slide is dipped into the precursor solution and dried over a hot plate at 240 °C to evaporate the solvent and partially remove surfactants such as oleyl amine and octadecylamine. The FTO glass slide is cleaned in soap water and then treated with acetone to remove any surface dust. The dip coating is made with only one dip into the solution, while the thin film is made through a detailed optimization study. After drying, the coated film is heat-treated in a muffle furnace (carbolite) in a non-isothermal manner at temperatures ranging from 470 °C to 550 °C at a ramping rate of 2 °C min^−1^. This condition is found by optimizing the process parameters. To obtain Mo doped BiVO_4_ thin films, the following optimization conditions are used. The number of layers deposited for the thickness-dependent study ranges from one to nine, with a heat treatment temperature range of 450 °C to 650 °C. The heat treatment dwelling time (heat exposure time inside the furnace) is set for 30 minutes, one hour, two hours, and four hours, with a ramping rate of two degrees Celsius per minute. Finally, the heat treatment temperature is optimized for 315 °C for 26 minutes; 393–471 °C for 19 minutes; and 472–550 °C for 26 minutes at a rate of 3 °C min^−1^. The best photocurrent density from a specific film made at a specific condition is used to determine the optimization condition. This is followed by optimization of the surfactant's combinatorial choice to inorganic and organometallic salts, and Mo-doping concentration is optimized for different amounts of molybdenyl acetylacetonate to Bi and V source compounds.

## Experimental

### Mo-doped BiVO_4_ film characterization

The optimized films are then characterized under different conditions to investigate their optical, structural, morphological, and photoelectrochemical functionality. An ALS Inc. SPEC 2000 spectra system is used to collect the UV-Vis spectra for optical property characterization. The data are then further analysed to determine the energy band gap of Mo doped BiVO_4_ film. All optimized film crystallographic properties are investigated using a Brucker D8 X-ray diffractometer, followed by post photoelectrochemical treatment of the best-optimized films. A CuK_α_ radiation source is used for the measurement using the LynxEye detector. In normal angle mode, the XRD is measured from 20 to 80° Bragg angles. The optimized film morphology is studied using a Zeiss ULTRA 55 digital field emission scanning electron microscope (FE-SEM) [courtesy of the FIRST Center at ETH Zurich]. The films were mapped using an EDX detector on a Leo 1530 Gemini field emission scanning electron microscope (FE-SEM). To improve the conductivity of some films before imaging, and Au–Pd sputtered coating was applied. A Bal-tec SCD 050 sputter coater was used. The thickness of the films is measured using a Bruker Dektak XT profilometer. Before measurement, the films are scratched with a knife to define the edge profile. Scanning transmission electron microscopy (STEM) images and elemental maps were obtained using an FEI Talos F200X microscope (200 kV). The microscope is equipped with a high-brightness Schottky FEG as well as the Super-X integrated EDS system, which included four symmetrically arranged silicon drift detectors (SDDs) around the sample. 200 kilovolts (kV) is the accelerating voltage. Superimposing a stack of images taken at each successive time interval in a time-domain resulted in the video TEM. XPS was performed in constant analyser energy (CAE) mode using a Sigma II Probe instrument (Thermo Fisher Scientific) equipped with a UHV chamber and a non-monochromatic 200 W Al K_α_ source (*hν* = 1486.6 eV). The source and emission angles were set to 50 degrees and 0 degrees, respectively. The pass energy was set to 25 eV with a step size of 0.1 eV. Casa XPS software was used to create all data analyses and fittings. Binding energies were corrected by adjusting the carbon (C1s) peak to 284.8 eV. The Shirley algorithm^1^ was used for background signal subtraction, and pseudo-Voigt line shapes were used for peak fitting.^1^ D. A. Shirley, *Phys. Rev. B*, 1972, **5**, 4709–4714.

### Photoelectrochemical and IPCE measurement

After the characterization of the optimized films, we have measured the photoelectrochemical functionality with a Volta lab 80 potentiostat in a three-electrode configuration using Ag/AgCl (3M KCl) as a reference electrode and Pt as the counter electrode. The cell used for this purpose is called as “cappuccino cell”. The total geometric area of the electrode immersed in the electrolyte is 1 cm^2^ and light-exposed areas are 1.50 cm^2^ and 0.7 cm^2^. The light source used for photocurrent density measurement is an AM1.5Xe light source from LOT Oriel, Switzerland, equipped with an electronic shutter operated manually and is used for the transient photocurrent density measurement. The electrolyte used for this purpose is 0.1 M phosphate buffer with pH = 7. The best-optimized films are photoelectrochemically measured in the presence of glycerol (20% V/V with 0.1 M phosphate buffer without diluting) to see the glycerol oxidation process. Here, glycerol is thoroughly mixed with the electrolyte solution before exposing it to light. Afterwards, the IPCE is measured with a Monochromatic light source from LOT oriel equipped on a Volta lab 40 potentiostat. The stability of the electrodes under light and dark conditions was studied in both phosphate buffer and phosphate buffer plus glycerol applying chronoamperometry methods with a fixed potential of 1.6 V *vs.* the Ag/AgCl electrode. Impedance data are collected in EIS mode by applying a fixed potential frequency range of 1 MHz to 1 Hz.

#### *Operando* gas chromatography measurement

Photoelectrochemical glycerol oxidation and the amount of hydrogen evolved were measured in a Plexiglas reactor equipped with a Voltalab 80 potentiostat and operando gas chromatography.^55^ The reference electrode was Ag/AgCl, and the counter electrode was Pt. To monitor the gases evolved from the water-splitting reaction and glycerol conversion into CO_2_ into the headspace of the three-electrode Plexiglas cell, a closed cycle, the recirculating gas mode is used. The electrochemical measurement was carried out using a chronoamperometry measurement with a fixed bias of 1.6 V *vs.* Ag/AgCl (3.5 M KCl). The aliquot from the GC experiment is further characterized *via* a ^1^H NMR study (MAS NMR from Bruker, 400 MHz) to confirm the conversion of glycerol into formic acid and formaldehyde. For the liquid-state NMR, 5 mg of the sample was diluted in D_2_O and filled in a 5 mm NMR tube. Samples were analyzed using Avance III HD NMR (Bruker) at a central frequency of 400 MHz.

### Photodegradability study of Mo-BiVO_4_ films under continuous light exposure

The photo degradability of Mo doped BiVO_4_ thin films is investigated under AM1.5 light illumination in the presence of glycerol in a stepwise time-dependent LIGHT exposure experiment. The charge transfer characteristics of the films were investigated using a Gamry impedance analyser (Gamry interface 1000 potentiostat).

### UV-Vis experiment of Mo-doped BiVO_4_ under glycerol oxidation

The optical properties of Mo-doped BiVO_4_ film are studied operando regarding the applied potential synchronous with the chronoamperometry experiment run on a Gamry interface 1000 potentiostat. The optical UV-Vis set up SEC–2000 used for the measurement, which is equipped with a flow cell, was purchased from ALS Inc. Japan. The electrolyte is injected into the flow cell using a syringe, and the source and the detector are separated by an optical fibre cable, as shown in Fig. X. The electrochemical potential used ranges from OCP to 1.4 V.

## Conclusions

A Mo doped BiVO_4_ photoelectrode is synthesized by a novel surfactant mediated approach with a well controlled morphology and pure clinobisvanite phases as confirmed by FESEM and XRD. The photoelectrode shows the usual absorbance band in the visible region thus confirming its use for photoelectrochemical purposes. A photocurrent density of 3.5 mA cm^−2^ is exhibited when exposed *via* a full aperture in front light illumination and is the highest among the existing pristine bismuth vanadate photoanodes without any modification. The scratch nanoparticles constituting the photoelectrodes show a unique phenomenon of dynamic crystallite fringes upon TEM beam exposure thus signifying the photodecomposition nature of the photoelectrode. To avoid the electron hole recombination and improve the STH efficiency, Mo-BiVO_4_ is further applied for photoelectrochemical glycerol oxidation, and it shows the highest current density of 5 mA cm^−2^ upon back side illumination. Here, front light illumination shows a lower photocurrent density due to the photon obstruction by a glycerol layer on the photoelectrode surface. The photoelectrode shows a photocharging phenomenon, thus allowing the photocurrent density to reach a maximum value of 8 mA cm^−2^ with a water splitting current density of 5.8 mA cm^−2^. The effect is caused by the increased band bending at the photoelectrode surface which allowed improved charge generation thereby preventing the recombination of excitonic pairs. The photoelectrode finally shows significant hydrogen gas evolution with the highest STH efficiency of 5.5% and IPCE of 52% upon photoelectrochemical glycerol oxidation. The post-photoelectrochemically processed Mo-BiVO_4_ photoelectrode shows a variation in the respective parameters such as the Bi/V ratio and electronic structure and finally gives an idea about its photostable nature. The outlook for extending the photoelectrochemical biomass-based hydrogen evolution process could impact the biodiesel industry in the partial hydrogenation of biodiesel to make it oxidation resistant.

## Author contributions

D. K. B. designed, planned, and carried out these tasks, which were followed by the writing of the entire manuscript. He conducted all experiments, except for a few characterizations, and he proposed the TEM beam induced lattice dynamics concept. D. H. did the TEM and HAADF EDX mapping work, as well as the TEM beam exposure snapshot experiment and corresponding image to videography conversion. P. C. F. did the *ex situ* XPS characterization. M. N. conducted an NMR investigation of the electrolyte to verify biomass oxidation. A. A. performed SEM characterization as well as EDX mapping of thin films. R. T. carried out the operando GC experiment and analysis.

## Conflicts of interest

There are no conflicts to declare.

## Supplementary Material

YA-001-D2YA00077F-s001

YA-001-D2YA00077F-s002
