# Supplementary material for: Photoelectrochemical glycerol oxidation on Mo-BiVO4 photoanodes shows high photocharging current density and enhanced H2 evolution
Source: Energy Adv. 2022 Aug 19;1(10):715–28. doi: 10.1039/d2ya00077f (PMC9558240; doi:10.1039/d2ya00077f)
Supplement: YA-001-D2YA00077F-s002 [file YA-001-D2YA00077F-s002.pdf]

Electronic Supplementary Material (ESI) for Energy Advances.
This journal is © The Royal Society of Chemistry 2022

Supporting information

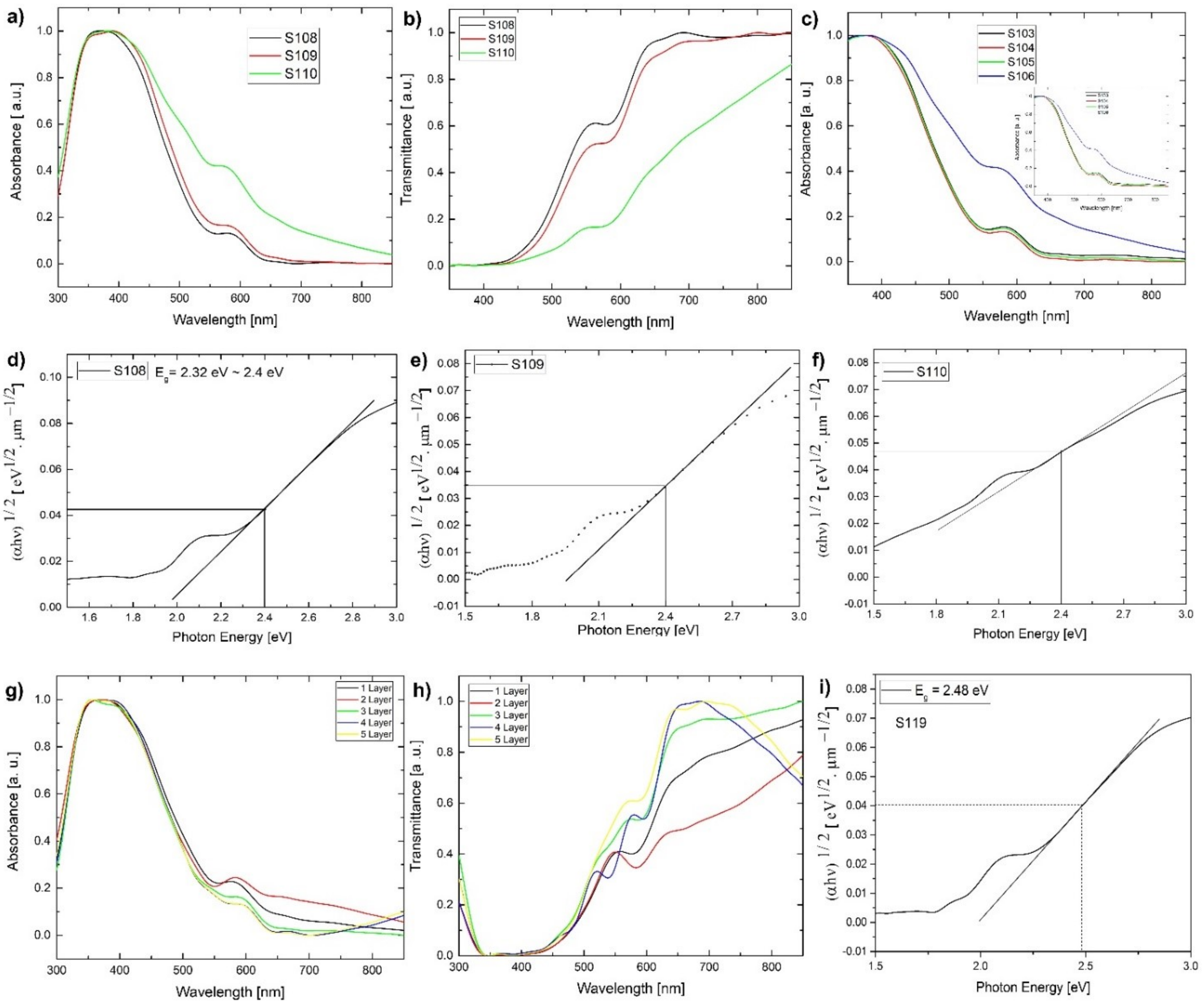

Figure S1: a) - b) UV-Vis absorbance and transmittance spectrums of Mo-$BiVO_4$ samples optimized for different annealing temperatures; c) UV-Vis absorbance spectrum of Mo-$BiVO_4$ samples made with different dwelling time; d) - f) Energy band gap calculation using Tauc method for Mo-$BiVO_4$ samples optimized at different annealing temperatures; g) -h): UV-Vis absorbance and transmittance spectrums of Mo-$BiVO_4$ samples optimized for different film thicknesses; i) Energy band gap calculation for Mo- doped $BiVO_4$.

Discussion: The absorbance properties of annealed temperature-dependent samples are also investigated. The absorbance and transmittance spectrums, as shown in figure S1, show that the

high dwelling time exposed samples have a significant increase in the intensity of the absorption band at 600 nm. In addition, the absorbance increased sequentially for samples S108 and S109. This cannot be directly correlated to any changes in the electronic structure of the films, as the bandgap calculation shows that it remains constant in each case. The change in transmittance in each case in the transmittance spectrum indicates the effect of increasing the thickness of the Mo doped $BiVO_4$ films on the photoabsorption behaviour. The absorption intensity near the visible light active band at 590 nm of doping dependent Mo doped $BiVO_4$ photoanode changes. However, bandgap calculations do not reveal any significant differences, which are 2.5 eV and correspond to the standard band gap value of $BiVO_4$. As a result, we must again correlate such changes in the inhomogeneous thickness distribution. Visual inspection indicates that the optimized films are not homogeneous in nature.

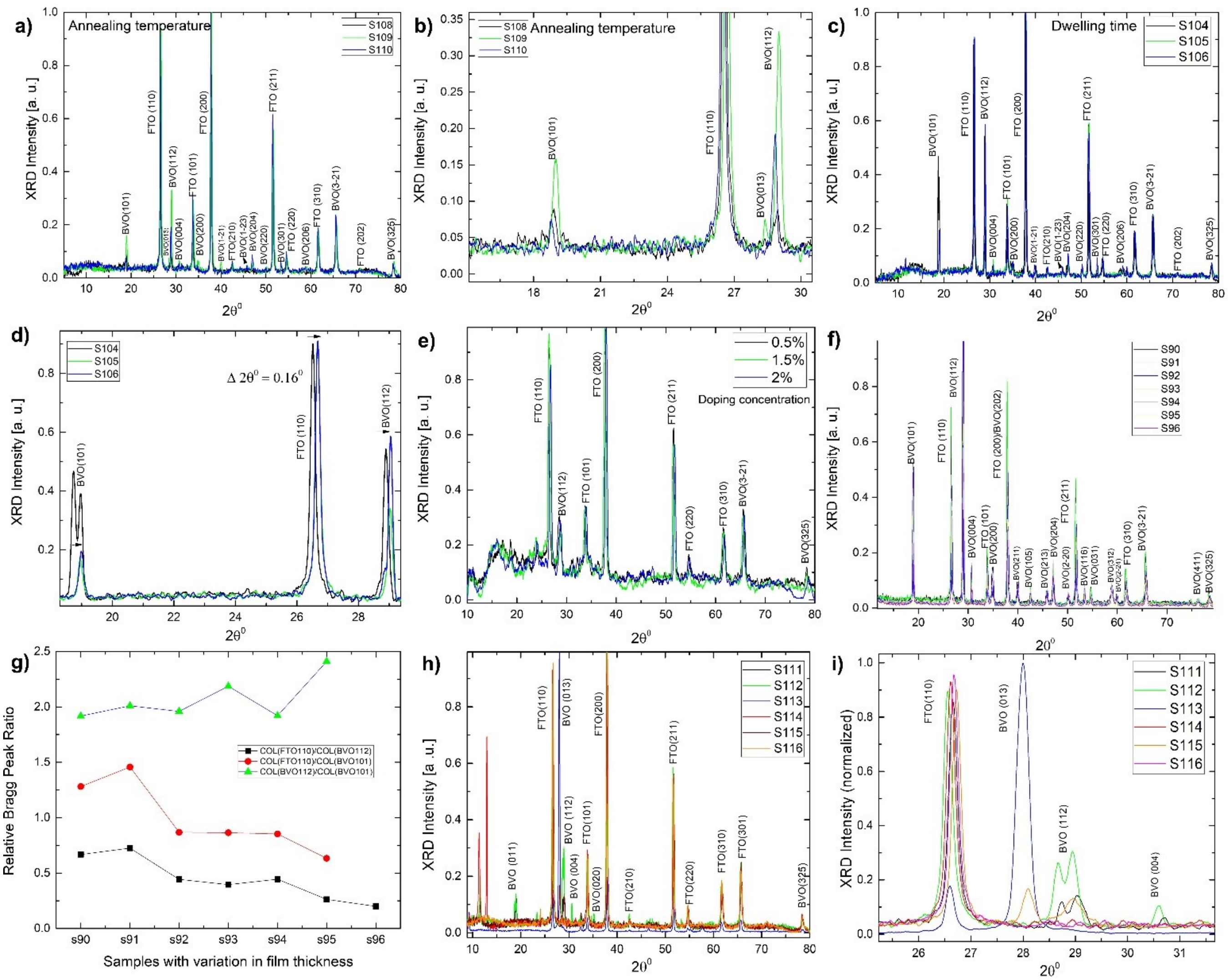

Figure S2: a)- b) Thin-film X-ray diffractograms of Mo-$BiVO_4$ samples optimized for different annealing temperatures and enlarged X-ray diffractograms shows the evolution of (101) peaks. c) - d) Thin-film X-ray diffractograms of Mo-$BiVO_4$ samples optimized for different annealing temperatures and enlarged X-ray diffractograms show the peak – splitting of (101) and (112) planes for low dwelling temperatures; e) Thin-film X-ray diffractograms of Mo-$BiVO_4$ samples optimized for different doping concentration; f) Thin-film X-ray diffractograms of Mo-$BiVO_4$ samples optimized for different layer thickness; g) Variation of relative Bragg peak ratio with samples with film thickness variation; h) - i) Thin-film X-ray diffractograms of Mo-$BiVO_4$ samples optimized for different precursor synthesis and expanded X-ray diffractograms showing the variation of Bragg peaks.

Discussion: The structural properties of as-prepared BiVO4 photoelectrodes are investigated for various optimization parameters such as annealing temperature, doping concentration, and dwell time. The shape of the Bragg diffraction peak changes significantly when Mo doped BiVO4 electrodes are made with different dwelling times. The expanded XRD profile clearly shows that the Bragg peak shifted to the higher Bragg angle, though a direct correlation cannot be established. The S-104 peak (101 planes) shape has convoluted into a single peak, with a slight shift in the Bragg diffraction angle for the (112) plane [Figure S2 a) - d] All of the diffractograms for the 0.5 per cent, 1.5 per cent, and 2 per cent Mo- doped samples remain unchanged from the doping dependent study. Figure S2 f)-g) shows that the crystallographic properties of thickness optimized films do not show any significant changes in the X-Ray diffractograms. We analyzed the changes in the Bragg peak diffraction pattern intensities of respective Mo-doped $BiVO_4$ crystallographic planes and plotted them against the number of layers deposited to see the variation in film thickness of different dip coated layers (3 – 9 layers). It is clear from the form that all of the planes (112), (104) and (213) evolve constantly, ruling out any crystallographic preferred growth. The relative Bragg peak ratio with substrate peak intensity exhibits a similar pattern, indicating non-crystallographic preferred growth. In the case of thickness optimized samples, the crystallite size increases abruptly, which is negligible in the case of nanostructured formation. The profilometric measurements of the various films reveal the following trend in the increase in film thickness.

Table S1:

Number of layer deposition	Thickness
1 Layer (S88)	90 nm
2 Layer (S 89)	2 µm
3 Layer (S90)	1.62 µm
4 Layer (S91)	1.78 µm
5 Layer (S92)	1.16 µm

Following that, we investigated the crystallographic properties of Mo-doped $BiVO_4$ films deposited from various precursors [Figure S2 h) – i). Precursors were optimized in a combinatorial approach to the presence and absence of the fatty acid stabilizer while keeping the salt and dopant ration constant. The goal is to observe the effect on the crystallite size and the effect on X-ray

diffractograms. The broad shape of the diffractograms for some cases S112 in comparison to S113 in terms of (112) and (013) planes confirms the crystallite's small size. The Scherrer equation was used to calculate the size of each crystallite, which is shown in the table below.

Table S2:

Sample (annealing temperature optimized)	Crystallite Size (nm)
S108	80 nm
S109	96 nm
S110	193 nm
Sample (Change in the type of precursor)	Crystallite Size (nm)
S111	23 nm
S112	29 nm
S113	80 nm
S115	33 nm

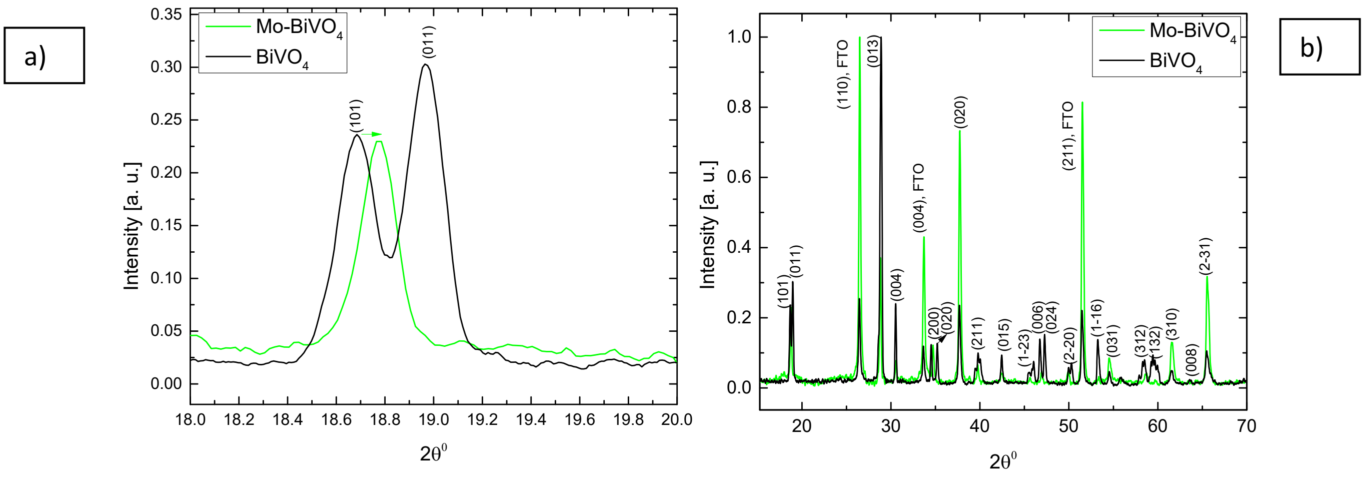

Figure S3: a) – b) Comparison of thin-film XRD profiles of pure $BiVO_4$ and Mo $BiVO_4$.

Discussion: On comparing the XRD of pure $BiVO_4$ with the doped one, it is found that the (101) peak shifted towards a higher Bragg angle on Molybdenum doping.

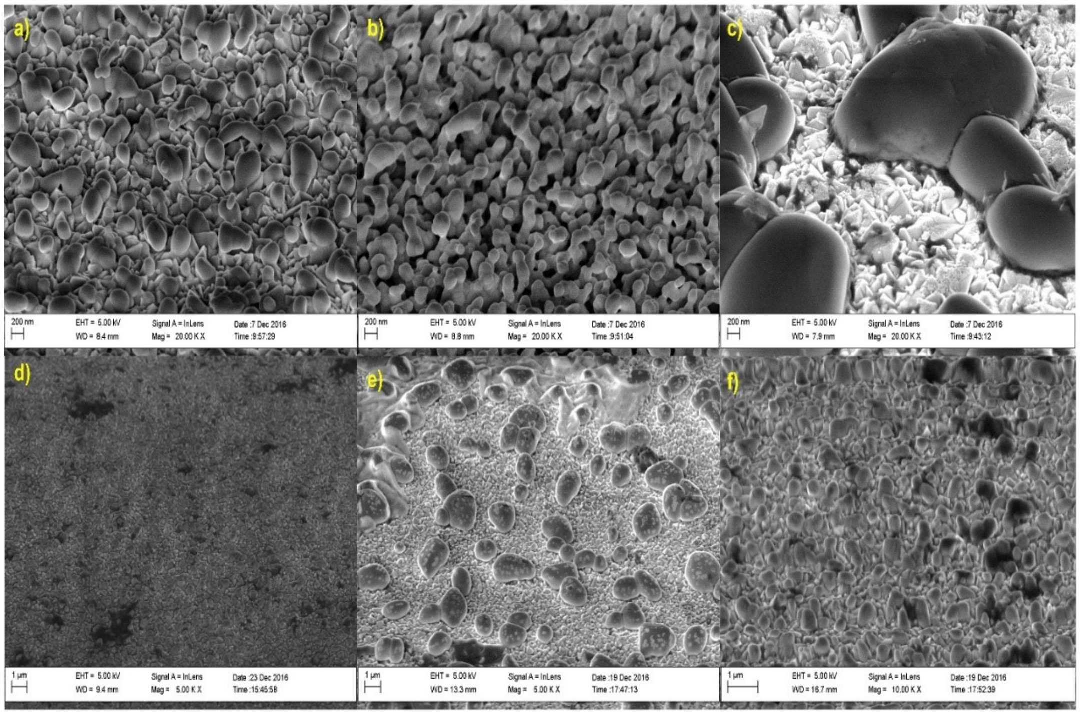

Figure S4: Field emission scanning electron micrographs (FESEM) of differently optimized Mo-$BiVO_4$ samples.

Field emission scanning electron microscopy is used to investigate the morphology of Mo doped $BiVO_4$ photoelectrodes under various optimized conditions [Figure S4]. According to the thickness optimized films morphology study, the sample S88 prepared in a non-isothermal heat treatment procedure with one-layer deposition exhibits isolated particles with hump-like character and a size of around 1µm. The particles are formed in an ordered manner on the FTO crystals, with the inter-necking of the particles visible. S 98 shows particles of around 50 nm in a 200 nm resolution FESEM image for another film prepared in the same manner with the only difference being the drying step. In this case, the particles are denser in nature. Dumbbell shape nanoparticles with metal oxide thin film morphology are observed here. The increase in the number of layers (3 layers) for S 100 reveals intriguingly similar "hump"-like characteristics. However, the only factor that makes sense, in this case, is that the inter necking phenomenon is more pronounced among

particles. The morphology of the dwelling time optimized films S 103–S106; all show well-distributed particles across the FTO substrate except S 105, which has very large particles. This is obvious because more dwell time inside the furnace causes particle agglomeration compared to less dwelled samples. For example, dwelled samples of 30 minutes (S103) and 1 hour (S104) do not show such particles. In the case of close imaging of sample S 104, we made one interesting observation. The sample contains a large translucent particle with small particles inside it. The mechanism of formation is beyond the scope of this discussion. The annealing temperature optimized samples, S 108, do not exhibit any particle formation characteristics. It's obvious because the temperature of $BiVO_4$ nucleation begins at 480°C.

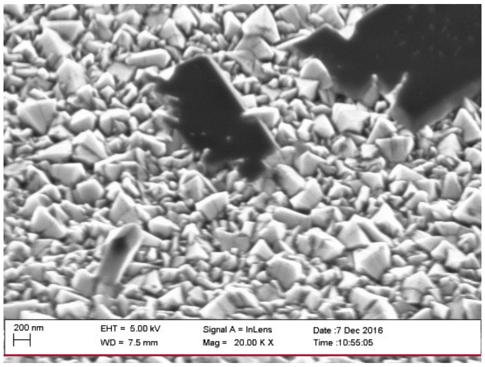

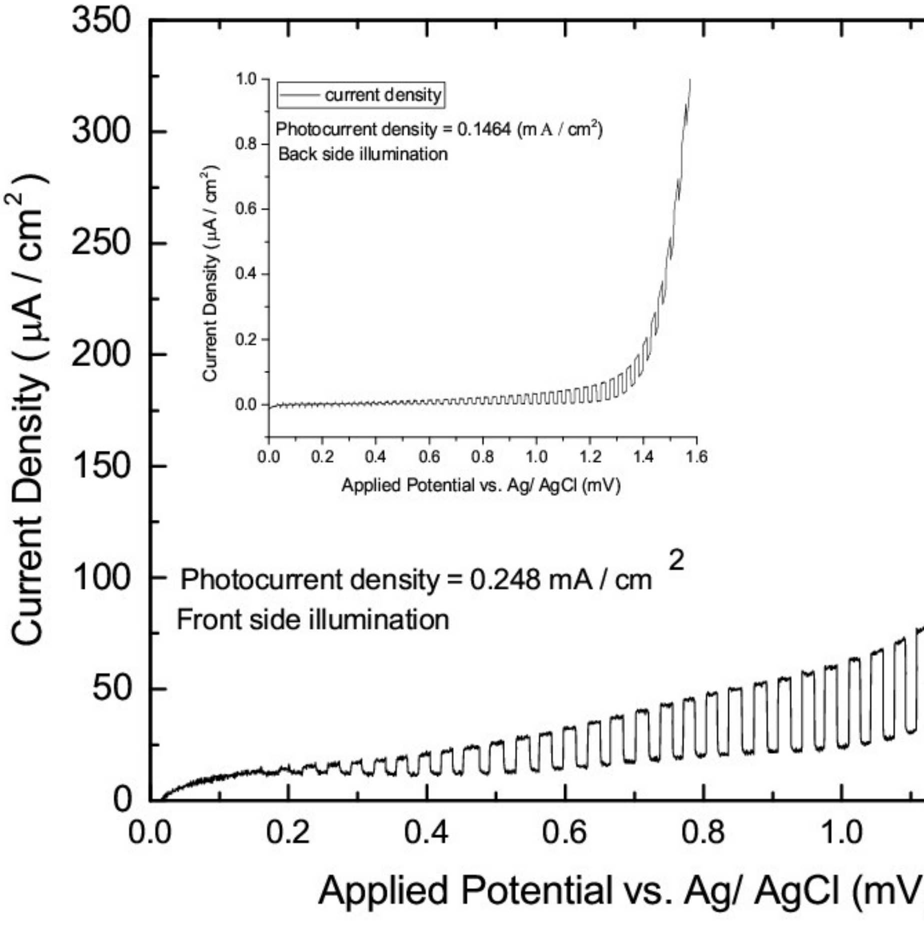

Figure S5: Transient photocurrent density measurement of pristine $BiVO_4$ photoelectrode.

For the photoelectrochemical measurements, we first tested the photoelectrode S75, which was made by direct dip coating of the obtained precursor on FTO. The photocurrent obtained for this

electrode is 248 μA / cm^2 when measured from the front side AM 1.5 light illumination, while the photocurrent density obtained from the backside illumination is 146 μA / cm^2. It should be noted that this is the first set of films that have not been optimized in terms of heat treatment temperature and dwell time. The total heat treatment time is 3 hours, with the following sequence: 450°C - 650°C for 1-hour ramping at a rate of 3°C/min, followed by dwelling at 650°C for 2 hours, and cooling from 650°C - 450°C for 2 hours. The film is transparent, and the colour is yellow with a greenish tint. The film was removed from the furnace at 450°C and allowed to cool inside a fume hood until it reached room temperature before being measured for photocurrent.

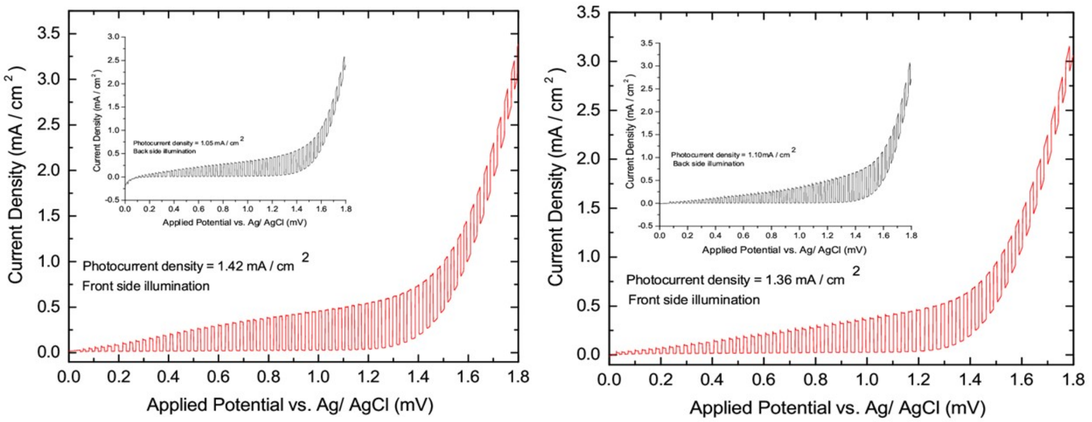

Figure S6: Transient photocurrent density measurement of Mo-$BiVO_4$ photoelectrode optimized for different experimental conditions.

Following that, we measured the photocurrent densities of photoanodes S76 and S77. At 1.4 V, reasonable total photocurrent densities of 1.42 mA/cm^2 and 1.36 mA/cm^2 are obtained in both cases. It exhibits the same photocurrent densities of 1 mA / cm^2 under the front and back illumination. When the water splitting potential is taken into account, the photocurrent density of S76 at 0.6 V for 0.1 M phosphate buffer (pH =7) electrolyte is 250μA/cm^2. In a few experimental conditions, the films used in this case differ. They are described in this section. Dip coating of the supernatant obtained from the centrifugation of the precursor was used to create the film S76. Please see the materials and methods section for more information on the dip-coating procedure. The drying step used to make the film is not used here, whereas the film S77 was dried at 120C on a hot plate before heat treatment. The same heat treatment procedure as for S75 is used here. The photoelectrode's light-exposed geometrical area is 1.5 cm2. When CV is applied from -1.8 V to +1.8 V, the photoelectrode S77 is completely corroded with no sign of photocurrent. The photoanode S76's transient photocurrent density shows only anodic spike formation. This indicates that electron-hole recombination occurs in bulk, whereas the absence of a cathodic spike indicates that no electron-hole recombination occurs at the photoelectrode's surface [1].

1. Dotan, H.; Sivula, K.; Grätzel, M.; Rothschild, A.; Warren, S. C. Probing the photoelectrochemical properties of Hematite (α-Fe_2O_3) electrodes using hydrogen peroxide as a hole scavenger. *Energy. Environ. Sci.* **2011,** 4(3), 958-964.

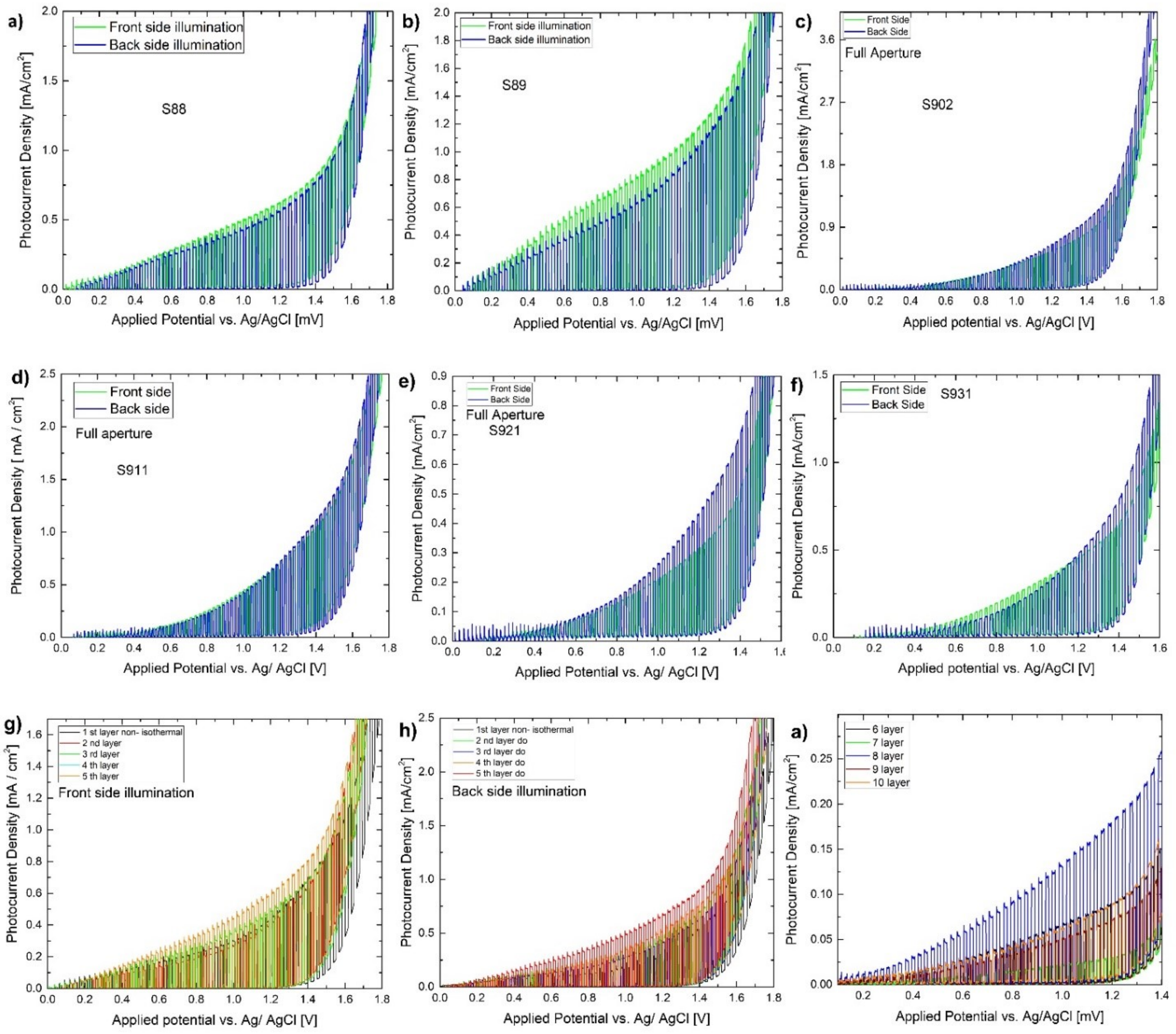

Figure S7: Transient photocurrent density of Mo-$BiVO_4$ photoelectrode optimized for different film thicknesses.

Discussion: The isothermal heat treatment of the precursor coated FTO up to 10-layer deposition is used to optimize the thickness of the Mo doped $BiVO_4$ electrode [sample S78 – S87], as shown in figure S7. The heat treatment temperature ranged from 450°C to 650°C, with subsequent cooling to 450°C. The deposited film, however, was not stable, as evidenced by the following SEM imaging [S84]. It does not demonstrate a promising photocurrent density. As a result, we used the non-isothermal thickness optimization method with a fixed temperature of 650°C. For thickness optimization, we used the following steps: Before the next layer deposition, an FTO substrate is dip-coated (manually) once and heat-treated at the specified temperature for 15 minutes before

being cooled outside. The same sequence is used in the hierarchical increase in layer thickness for the subsequent films [S88 – S97]. It should be noted that the photoactive materials completely cover the substrate FTO. The precursor coated FTO substrates are not dried at 250°C for S88 and S89 before the heat treatment step, as is done for the next set of thickness-optimized films [S98 – S102]. In the following case, the films are not splattered with photoactive materials. The photoelectrochemical measurement of the thickness optimized films is performed using linear sweep voltammetry in 0.1 M phosphate buffer within the potential window 0 to 1.8 V vs. Ag/AgCl. Chopped current measurements are also performed to better understand surface and bulk electron-hole recombination. The photocurrent density of the one-layer coated film S88 is visible under both front and backside illumination.

The photocurrent density of the next set of films, 2 layers coated [S89], is 1.2 mA/cm^2. The films [S90 - S96] demonstrate the variation in photocurrent density as thickness increases. For full AM1.5 light exposure, S-93 has a maximum photocurrent density of 2mA/cm^2. With 0.7cm^2 geometrical area exposure, sample S-93 exhibits nearly half of the current density. It also exhibits the characteristics of gas bubble evolution. As shown by the SEM images, the high photocurrent density is due to the good contacts of Mo-doped $BiVO_4$ beads with the FTO surface. The fact that the beads are well separated from one another could explain the increase in current density. The photocurrent density decreases as the film thickness increases, confirming the effect of increased absorption cross-section on the photocurrent density, which is followed by higher electron-hole recombination. Full cyclic voltammetry is used to measure the film S – 96 from -0.5V to 1.8V. During the cathodic scan, an interesting result was observed. The cathodic current density is greater here, measuring - 20 mA / cm^2. Given that, a control measurement was also performed with only the Ti clip to see if there was any parasitic current density from the Ti clip due to its exposure to the electrolyte. It is, however, negative in terms of the stated current density. The photocurrent density of the non-isothermal film set [S98] with no spillover of photoactive materials is 0.718 mA/cm^2. In terms of photocurrent density, the rest of the film exhibits a similar trend [S99–S102]. In both front and backside illumination, all samples exhibit pleasing photocurrent density trends. The spill over the sample, i.e. the sample coated on the entire surface of the FTO, is advantageous due to the formation of more isolated nanoparticles everywhere. It is significant from the standpoint of the device. It contributes to the light-harvesting action of each $BiVO_4$ "Nanoisland." The photocurrent density trend for the thickness-optimized film should show a rise as a "Gaussian

Distribution." Each incremental layer is subjected to heat treatment for X number of time thickness T. For example, a four-layer coated film was exposed to a 55-minute heat treatment time. This eventually increases the size of the Mo doped BiVO4 particles that comprise the film, as demonstrated by the crystallite size calculation from the XRD profile using the Scherrer formul**a.**

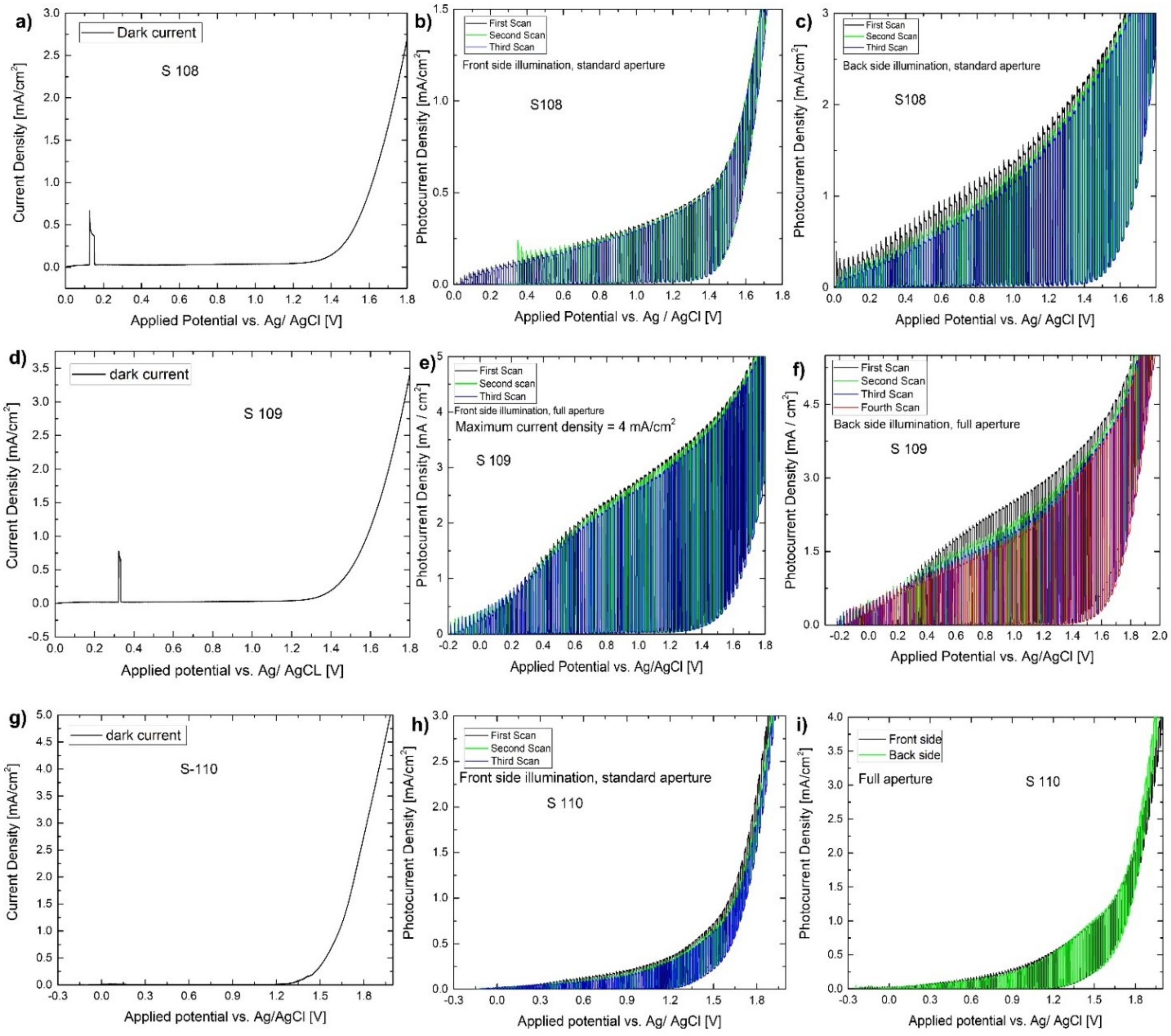

Figure S8: Transient photocurrent density of Mo-$BiVO_4$ photoelectrode optimized for heat treatment temperatures.

Discussion: Within the potential windows of +1.5 V to -1.5 V, LSV for sample S107 shows a reasonably higher dark cathodic current density. It should be noted that this particular sample was heat-treated from 320°C to 400°C, and the organic precursor was left on the film, as evidenced by its blackish appearance. The photocurrent density of sample S108 (heat-treated from 400°C to

470°C) is 500μA/cm^2 at 1.4 V before the onset of the Faradaic dark current, and it is measured with a standard light-exposed aperture size of 0.6 cm^2 under front-side illumination. To ensure reproducibility, each chopped photocurrent density is measured three times on average. When the light is exposed to the full aperture size of 1 cm^2, the photocurrent density rises to 1.4 mA/cm^2. In this case, we've normalized the photocurrent density based on the size of the light-exposed aperture. Following that, backside illumination yields a photocurrent density of 2.13 mA/cm^2. The dark current density of the LSV is 2.6 mA/cm^2. The photocurrent density of sample S 110 (heat-treated at 550°C - 650°C) is 580 μA/cm^2 at 1.4 V under full aperture illumination on both the back and front sides. Based on these results, it is clear that the sample optimized for 470°C - 550°C, S109, has the highest photocurrent density.

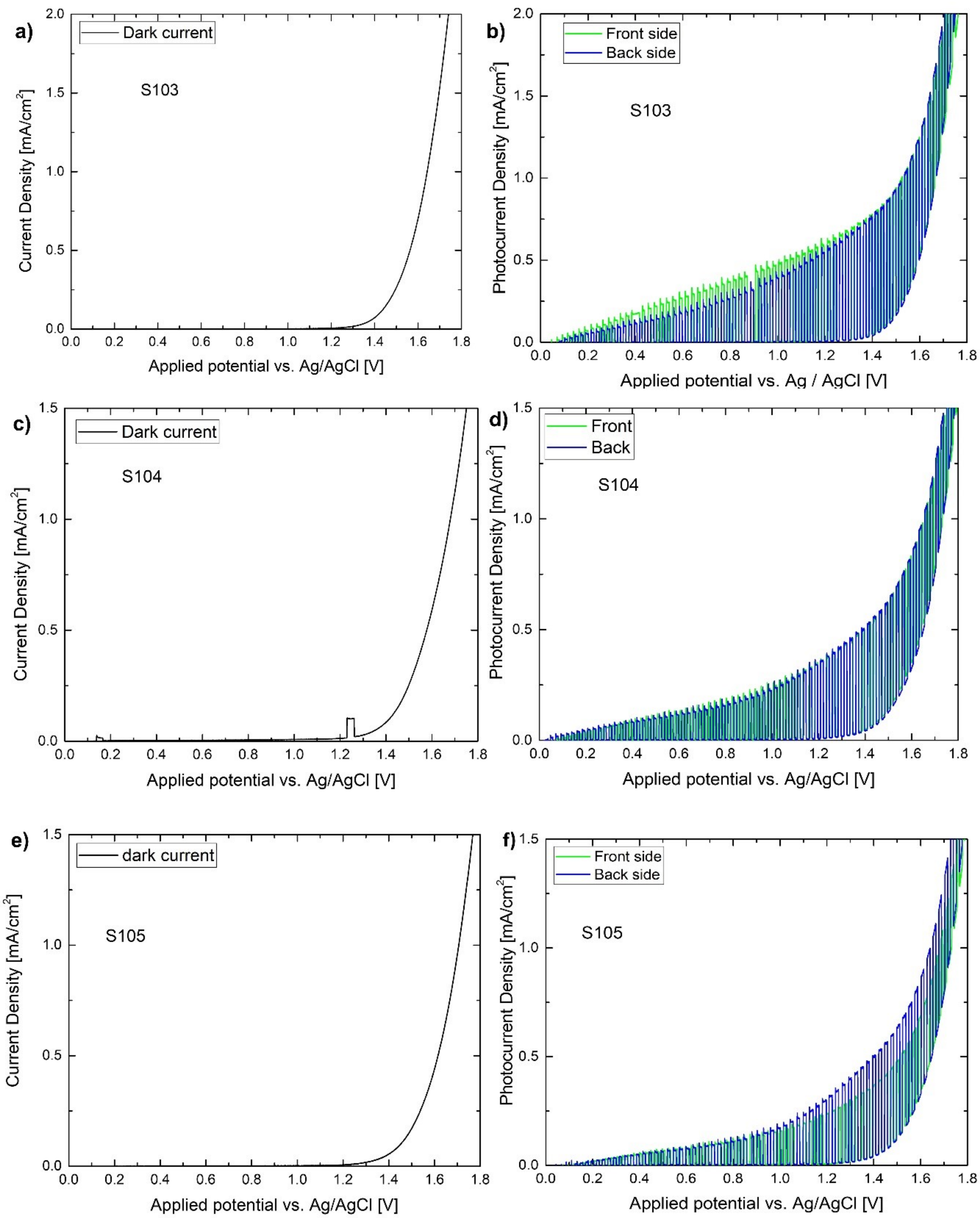

.

Figure S9: Transient photocurrent density of Mo-$BiVO_4$ photoelectrode optimized for different dwelling times during the heat treatment process.

Discussion: The photocurrent densities of optimized samples for various dwelling times are measured. On front side illumination, S103 (dwelling time = 30 min) exhibits a photocurrent density of 800μA/cm^2 at 1.4 V. The magnitude of photocurrent density is also visible on the backside illumination. At 1.6 V, the LSV has a dark current density of up to 2 mA/cm^2. On both front and backside illumination, the photocurrent density of S104 (dwelling time = 1 hour) is 520 μA/cm^2. For the AM 1.5 light exposure, the standard aperture size is used. The photoanode designed for a 2-hour dwell time (S105) has photocurrent densities of 366 μA/cm^2 and 485 μA/cm^2 under both front and backside illumination. The difference in photocurrent density is due to the photoactive layer (Mo-$BiVO_4$) being different from the current collector (FTO). Under front side illumination, the high dwelling time (4 hours) exposed sample S106 has a very low photocurrent density of 100 μA/cm^2. Based on the observations above, we decided to set the dwell time and heat treatment temperature for making Mo- $BiVO_4$ at 30 minutes and 470°C - 550°C, respectively.

1. **Effect of Mo doping (%) on the photocurrent density**

We also optimized the photocurrent densities of photoanodes by changing the dopant ratio (Molybdenum) from 0.5 per cent to 2 per cent, and all films were synthesized under previously optimized conditions [dwelling time = 30 minutes and heat treatment temperature 470°C - 550°C, with a ramping rate of 3°C/min]. We discovered that only 1% Mo-doped BiVO4 has a higher photocurrent density of 1.8 mA/cm^2 and 2.4 mA/cm^2 under the front and backside illumination with full AM1.5 light exposure. S-120 exhibits the same photocurrent density as S109 after being heat-treated for 30 minutes at temperatures ranging from 450°C to 550°C. The photocurrent density decreases dramatically when the temperature is increased from 550°C to 650°C at 3°C/min for all doping ratios, whereas non-isothermal annealing at 650°C for 15 minutes does not result in a good

\

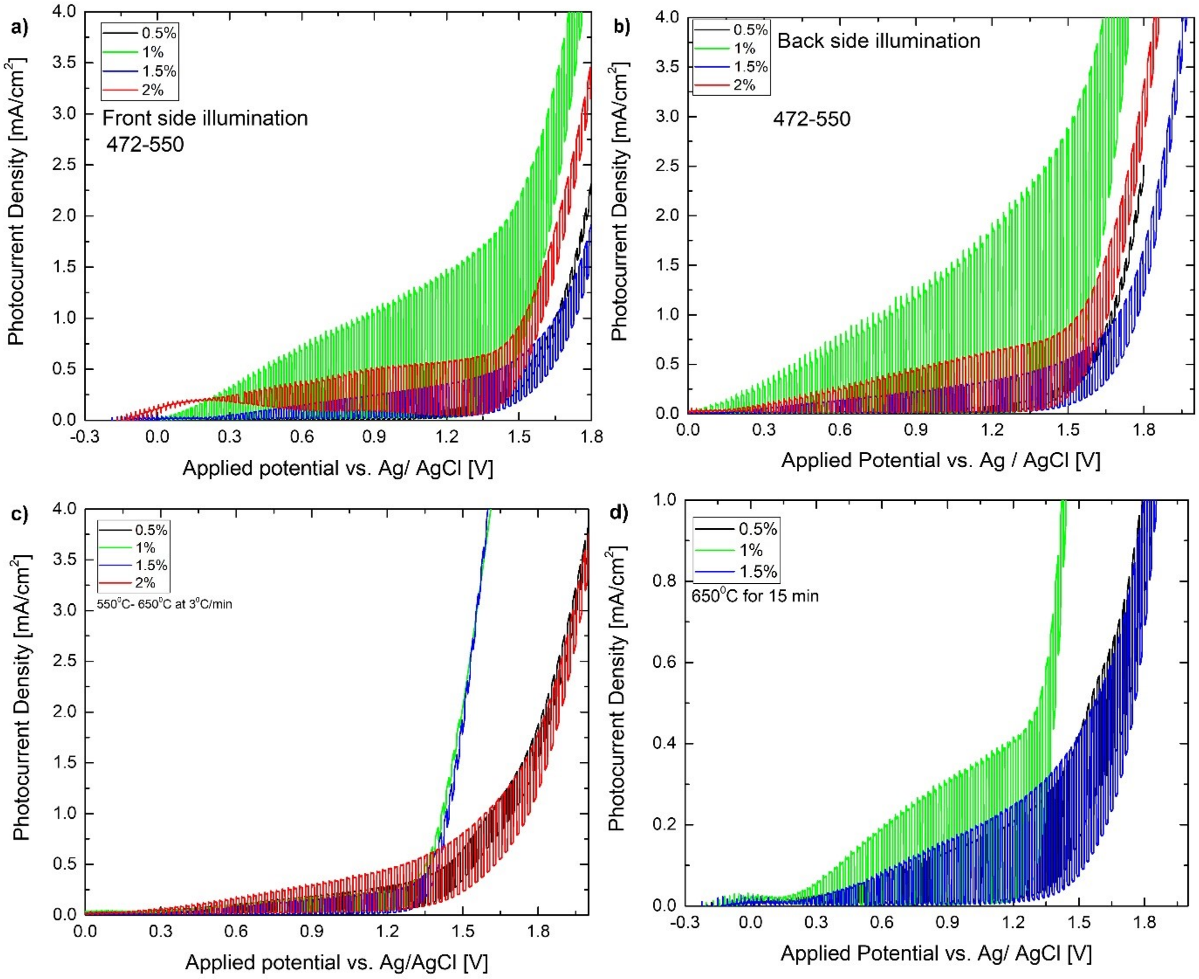

set of photocurrent density.

Figure S10: Transient photocurrent density of Mo-$BiVO_4$ photoelectrode optimized for different Mo - doping concentrations.

Table S3:

A) Front side illumination

Dopant ratio	Photocurrent densities
0.5% (S119)	0.2 mA/cm^2
1% (S120)	1.8 mA/cm^2
1.5% (S121)	0.5 mA/cm^2
2% (S122)	0.7 mA/cm^2

B) Backside illumination

Dopant ratio	Photocurrent densities
0.5% (S119)	0.18 mA/cm^2
1% (S120)	2.4 mA/cm^2
1.5% (S121)	0.45 mA/cm^2
2% (S122)	0.70 A/cm^2

2. Effect of precursor components on the respective photocurrent density

We also optimized the effect of the various components used to make the precursor on the photocurrent density. We optimized the precursors in terms of the presence and absence of oleic acid, oleyl amine, and octadecyl amine. First, we examine the case for the synthesis of $BiVO_4$ film without the use of Mo dopant but with all other fatty acid stabilizers (A). The precursor was then synthesized without the use of oleyl amine (B), oleic acid (C), and octadecylamine (D). Following that, we performed the second set of optimizations [E, F, G]. In this case, 15 mL of oleylamime is placed in a beaker and heated to 125°C. Then, 1.35 g of $Bi(NO_3)_3.5H_2O$ is dissolved in dried ethanol and stirred for enough time to form a white suspension. The suspension is then poured into the beaker containing the oleyl amine and manually stirred with a glass rod. First, a cream-coloured suspension with moderate bubbling is obtained, which may be due to the presence of water in dried ethanol. After the bubbling stops, the temperature of the reaction bath is gradually raised to 150°C and held for 1 hour. The cream colour suspension is transformed into a brownish form, and then into a dark brown form. Following this, the temperature is raised to 210°C and the reaction is allowed to run for 2 hours. The temperature was then raised to 270°C, and the reaction was repeated until all of the bubble evolution stopped. Before using these precursors to create the $BiVO_4$ film, the reaction was worked up by adding 40 ml of THF to the synthesized precursor mass and stirring until a clear solution was obtained. The suspension is then centrifuged at 7000 rpm for 7 minutes. The suspension is dip-coated with FTO and heat-treated at 8°C/min from 490°C to 590°C [S111]. The remaining films [S112–S118] are then heat-treated at 650°C for 15 minutes with a one-time dip coating. Because of the high ramping rate, the photocurrent density is low, on the order of 80µA/cm^2. Instead of 3°C/min, we used a faster ramping rate.

2.1. **Photoelectrochemical characterization of precursor optimized films:** open circuit potential is measured in the presence of light. Following that, linear sweep voltammetry is performed under chopped conditions between OCP and 1.8 V at a scan rate of 50 mV/sec. The electrolyte is 0.1 M phosphate buffer with a pH of 7. The cappuccino cell's black cap is removed to expose the entire electrode surface to AM 1.5 sunlight. S111, which was made by isothermal heat treatment from 490°C to 590°C for 8 minutes, has a very low photocurrent density when compared to S-109, which was made by the same procedure but with Mo doping. S112 has the same photocurrent density as S111. The photocurrent in the precursor B-made film S113 confirms the presence of the $BiVO_4$ phase. S114, which is made from precursor C, does not exhibit any photocurrent, whereas S115 exhibits photocurrent density in the same way that S112. Film S116 has a reasonable photocurrent density and film S117 has a good photocurrent density, but the magnitude of the spike transient photocurrent density increases as the bias is increased. S118 has no photocurrent density, and the spike is in both the anodic and cathodic directions, indicating bulk recombination.

2.2. **Replicability of processing conditions for obtaining the same order of photocurrent density:**

We created four distinct Mo-doped $BiVO_4$ films under the following conditions: [A. precursor coated FTO dried over the hot plate before heat treatment S143; B. no drying step before heat treatment S144; C. precursor coated FTO dried over a hot plate and cooled inside hood after heat treatment S145; D. without dried over the plate and cooled inside furnace S146]. The goal of creating these films is to replicate the processing conditions to obtain the most efficient Mo-doped $BiVO_4$ photoanode. The resulting films have a yellowish appearance and are heat-treated for 1 hour at 450°C - 550°C at 3°C/min. According to the photocurrent density measurements, all films have a similar amount of photocurrent density under both front and backside illumination, regardless of whether they were dried over a hot plate or not before the heat treatment step. As a result, we can confirm that the heat treatment sequence from 450°C to 550° C at a ramping rate of 3°C/min for 1 hour is the best-optimized condition for producing Mo-doped $BiVO_4$ films.

3. **Effect of front and backside illumination on the photocurrent density of Mo-BiVO4 photoanode with glycerol**

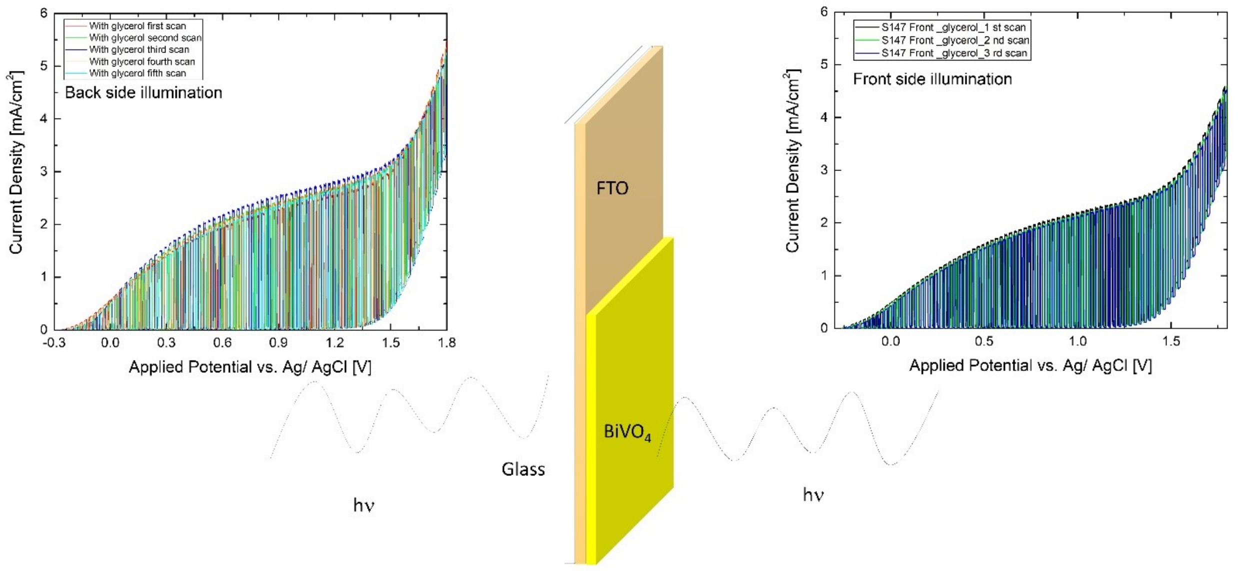

Figure S12: Transient photocurrent density of Mo-doped BiVO4 in glycerol under both back and front side illumination.

To further this, we synthesized four sets of Mo-doped $BiVO_4$ films (S147–S150) under optimized conditions. S147 - S150 Synthesis: FTO glass is cleaned in soap water + acetone before being dipped in Bi-precursor solution. Four sets of films (S147-S150) are dip-coated, and two of these are dried over a hot plate at 120°C, while the other two are heat-treated without any drying. The heat treatment sequence is as follows: 470°C - 550°C at 3°C/min for 30 minutes. It is worth noting that a 30 minute dwelling time results in the best photocurrent density performance. On front side illumination, the sample S-149 exhibits a photocurrent density of 2.23 mA/cm^2 at 1.2V, whereas back-side illumination has no discernible effect on the photocurrent density. The photocurrent density of sample S-147 is comparable to that of samples S149 - S150. In the presence of glycerol, the effect of front and backside illumination on the photocurrent density of a Mo-doped $BiVO_4$

photoelectrode is investigated. The photocurrent density for both cases is well matched. From this, it can be concluded that the photogenerated electrons and holes must travel the same distance from the electrode-electrolyte interface on both sides. The incident photons will have the same penetration length -1 in both cases, as explained elsewhere. [44]

It is hypothesized that defect-rich semiconductors absorb more glycerol, and research is being conducted to improve the efficiency of a materials design engineering effort (e.g. nanostructure and cationic doping). As a result of this, we have devised a strategy for developing doping enhanced efficient $BiVO_4$ semiconductors for this purpose. In previous work, recombination losses in the $BiVO_4$-based water photooxidation process were overcome by the use of overlayer and underlayer coated materials, which change the semiconductor surface physics to some extent. To explain the process, a hole mirroring phenomenon has been considered. SnO_2 acts as a hole mirror underlayer, preventing holes from migrating into the back contact. Because there are no recombination losses, the system's efficiency is greater than that of a pristine BiVO4 photoelectrode. This hole mirror also serves as a blocking layer for hole migration in gradient heterojunction-based photoanode. [44] Liang, Y., Tsubota, T., Mooij, L. P. A., & Van De Krol, R. (2011). Highly improved quantum efficiencies OF thin-film $BiVO_4$ photoanodes. *Journal of Physical Chemistry C*, *115*(35), 17594–17598. https://doi.org/10.1021/jp203004v

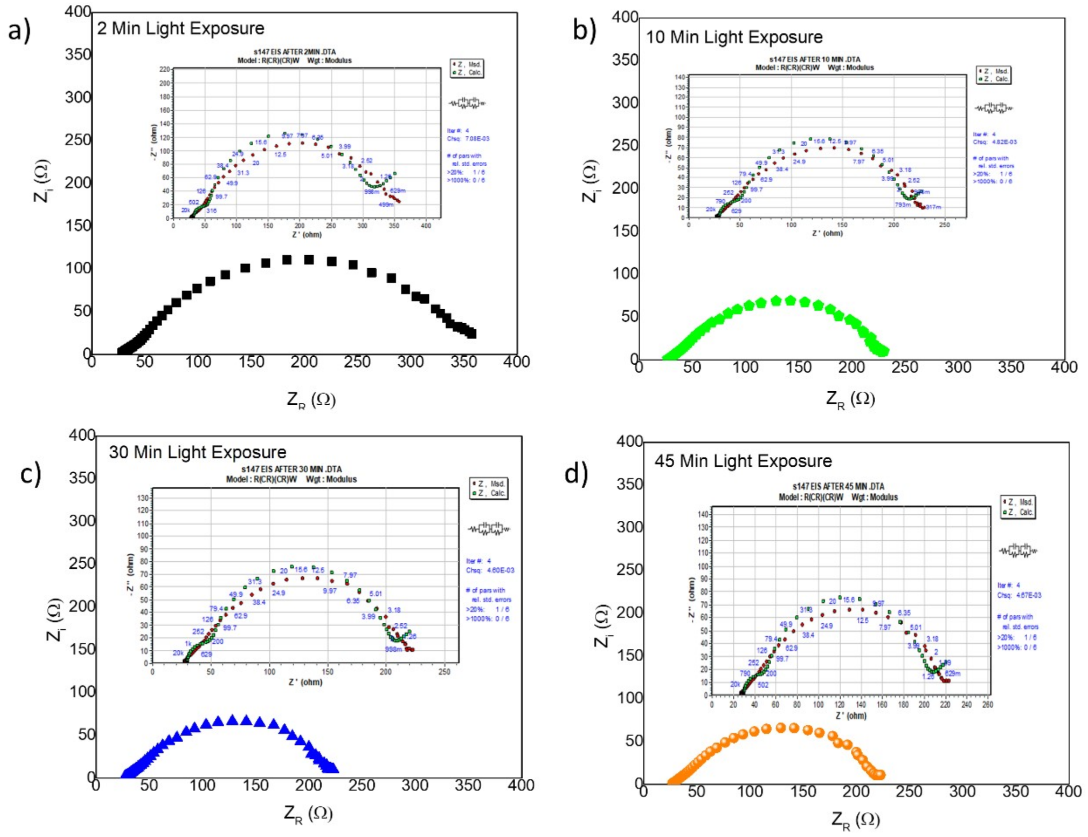

Figure S13: Electrochemical impedance data fitting using ZSimpWin software for a) 2 min b) 10 min c) 30 min and d) 45 min light exposure.

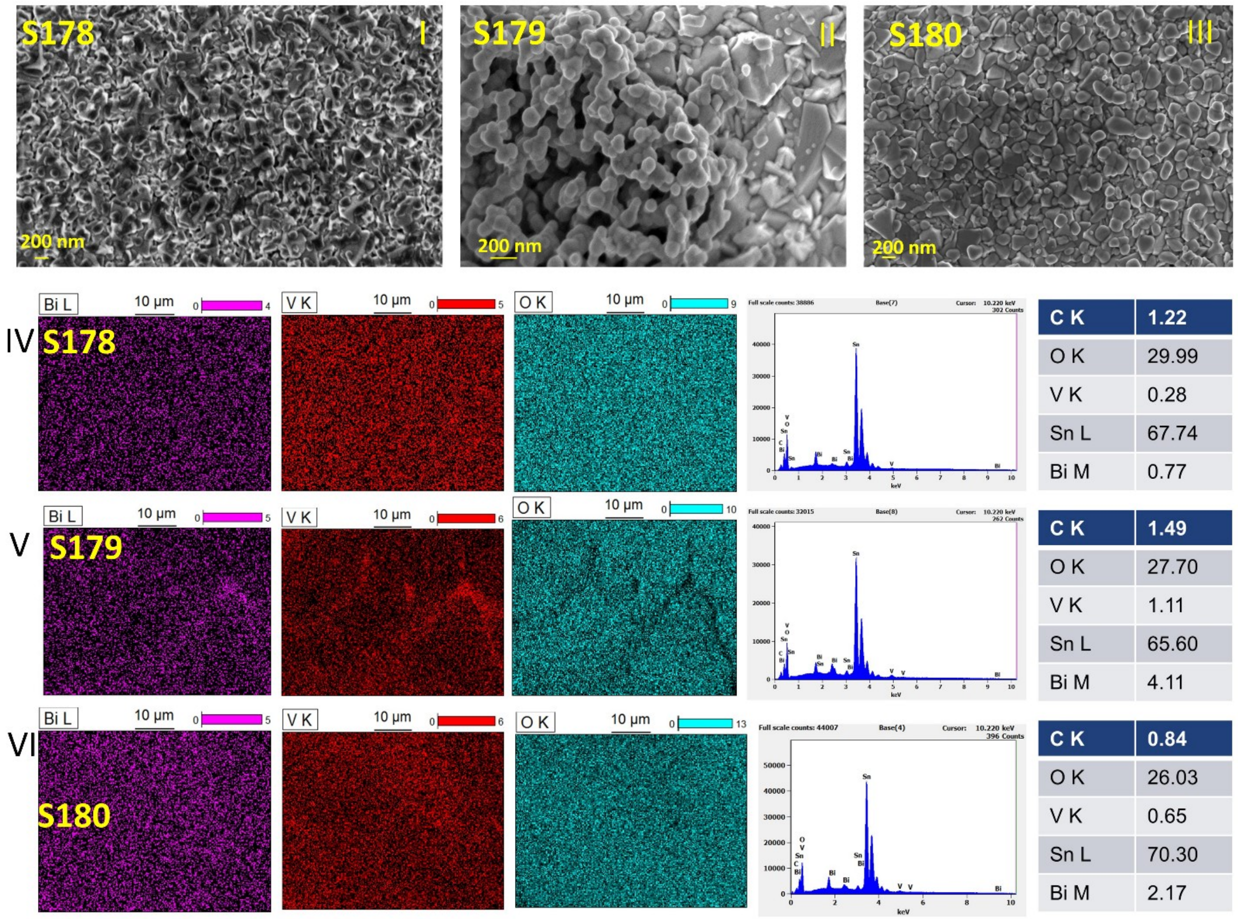

Figure S14: Ex-situ FESEM images of photoelectrochemically processed samples in the presence and absence of glycerol.

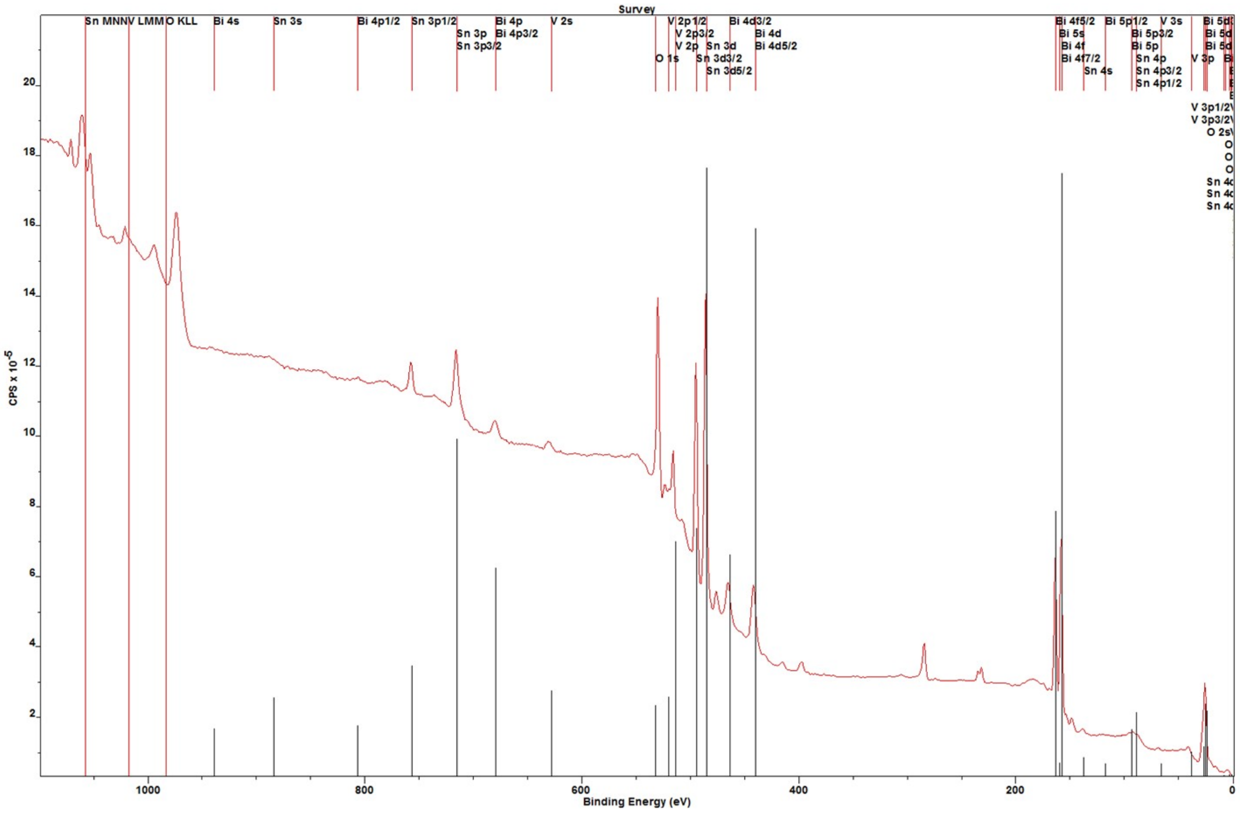

Figure S15: XPS survey scan of Mo-$BiVO_4$ photoanode.

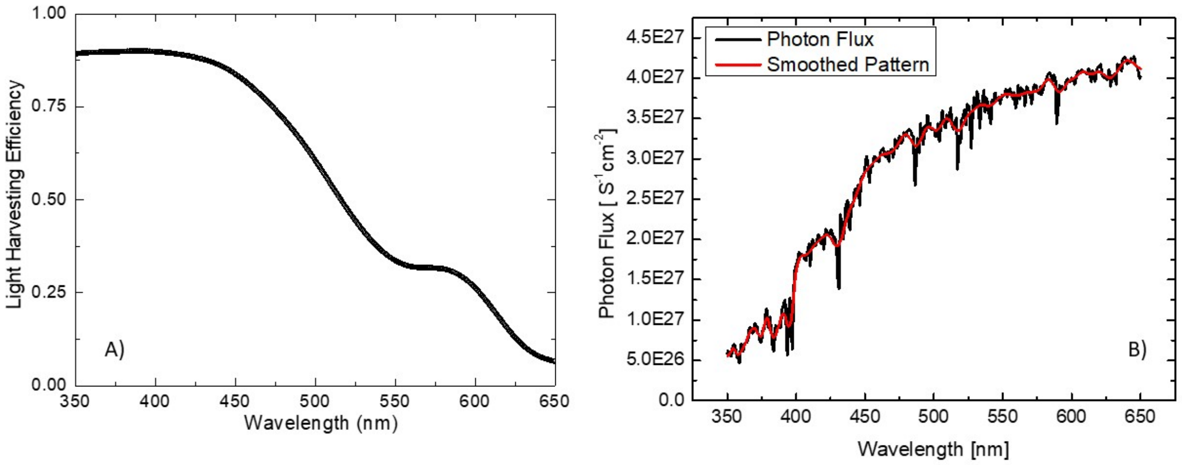

Figure S16: Light-harvesting efficiency calculation to wavelength; b) Variation of incident photon flux across the wavelength range.

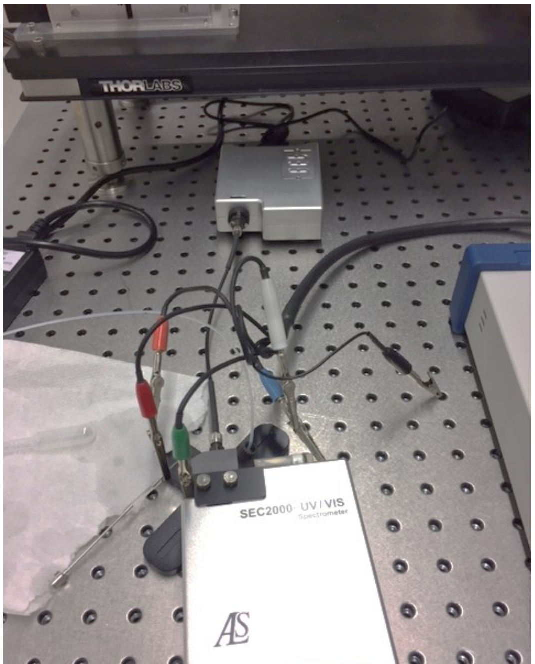

Figure S17: Opernado spectroelectrochemistry set up for studying the effect of applied potential on the light absorbance behaviour of Mo-$BiVO_4$ in the presence and absence of glycerol.

Table S4: Variation of V/ Bi ratio for Mo-BiVO4 sample evaluated by RBS .

Sample	V/Bi	
		±
S-195	0.89	0.02
S-194	0.93	0.02
S-193	0.94	0.02
S-192	0.66	0.04
S-191	0.95	0.06
S-190	0.95	0.03

Table S5: Ex-situ structural properties of Mo doped $BiVO_4$ film after glycerol oxidation are carried with Brucker D8 X-ray diffractometer. The following samples conditions are used for this experiment.

Sample	Experimental Condition
S-162	Pristine Mo-doped $BiVO_4$ film
S-163	Pristine Mo-doped $BiVO_4$ film+0.1M phosphate buffer
S-164	Pristine Mo-doped $BiVO_4$ film+0.1M phosphate buffer + 10% glycerol
S-165	CA 30 min at 1.4 V under dark, 0.1M phosphate buffer
S-166	CA 30 min at 1.4 V under light, 0.1 M phosphate buffer
S-167	CA 30 min at 1.4 V under dark, 0.1 M phosphate buffer + 10% glycerol
S-168	CA 30 min at 1.4 V under

	light, 0.1 M phosphate buffer + 10% glycerol
S-169	CA 30 min at 0.6 V under dark, 0.1 M phosphate buffer
S-170	CA 30 min at 0.6 V under light, 0.1 M phosphate buffer
S-171	CA 30 min at 0.6 V under dark, 0.1 M phosphate buffer + 10% glycerol
S-172	CA 30 min at 0.6 V under light, 0.1 M phosphate buffer + 10% glycerol

Table S6: Ex-situ morphology and bulk compositional study of Mo- doped $BiVO_4$ film with FESEM and Energy Dispersive X-ray spectroscopy (EDX): For the morphological study of the ex - situ photoelectrochemically treated films, Zeiss ULTRA 55 digital field emission scanning electron microscope (FE-SEM) is used [Courtesy of FIRST Center at ETH Zurich]. The EDX mapping of the films was carried out at Bi- L edge, V- Kedge and O K- edge respectively with Leo 1530 Gemini field emission scanning electron microscope (FE-SEM), which was equipped with an EDX detector.

S - 178	Pristine Mo-doped $BiVO_4$ film
S - 179	Pristine Mo-doped $BiVO_4$ film+0.1M phosphate buffer
S - 180	Pristine Mo-doped $BiVO_4$ film+0.1M phosphate buffer + 10% glycerol

Table S7: Ex-situ investigation of the electronic structure of $BiVO_4$ films with X-Ray photoelectron spectroscopy: XPS investigation is carried out for the following sets of samples.

….

S-184	Pristine Mo-doped $BiVO_4$ film
S-185	Pristine Mo-doped $BiVO_4$ film+0.1M phosphate buffer
S-186	Pristine Mo-doped $BiVO_4$ film+0.1M phosphate buffer + 10% glycerol

Table S8: Ex-situ investigations of the surface compositional properties with Rutherford backscattering experiment: The RBS characterization was carried out for the following sets of Mo doped BiVO4 films. Here ex-situ investigation is done by running a photocurrent voltammetry experiment before investigation. Samples have been analyzed by Rutherford Backscattering Spectrometry (RBS) [1] at the ETH Laboratory for Ion Beam Physics. Measurements have been performed using a 5 MeV ^4He beam and a silicon PIN diode detector under 168°. The collected data have been evaluated using the RUMP program [2].

[1] W.K. Chu, J.W. Mayer, M.A. Nicolet Backscattering Spectrometry, Academic Press, 1978.

[2] L.R. Doolittle, A semiautomatic algorithm for rutherford backscattering analysis, Nucl. Instr. and Meth. B15 (1986) 227.

S-191	Pristine Mo-doped $BiVO_4$ film
S-192	Pristine Mo-doped $BiVO_4$ film+0.1M phosphate buffer
S-193	Pristine Mo-doped $BiVO_4$ film+0.1M phosphate buffer + 10% glycerol

Table S9: Ex-situ Raman investigation of Mo–doped $BiVO_4$ films to probe change in the bonding state due to glycerol oxidation: The Raman investigation is carried out with a DXR™

2 Raman Microscope, Thermo Fisher. The excitation wavelength is chosen with a 455-nm laser. Following sets of samples are used for this investigation.

S-196	Pristine Mo-doped $BiVO_4$ film
S-197	Pristine Mo-doped $BiVO_4$ film+0.1M phosphate buffer
S-198	Pristine Mo-doped $BiVO_4$ film+0.1M phosphate buffer + 10% glycerol

7
